# SAA3 deficiency exacerbates intestinal fibrosis in DSS-induced IBD mouse model

**DOI:** 10.1038/s41420-025-02299-x

**Published:** 2025-01-26

**Authors:** Xiaodong Zou, Tong Wu, Jianjiao Lin, Tao Su, Hui Xiao, Chuyan Ni, Lijuan Hu, Wenchu Lin, Weilin Chen, Richard D. Ye, Li Xiang

**Affiliations:** 1 Department of Gastroenterology, The Second Affiliated Hospital, School of Medicine, The Chinese University of Hong Kong, Shenzhen & Longgang District People’s Hospital of Shenzhen, Shenzhen, 518172 China; 2 Guangdong Key Laboratory for Biomedical Measurements and Ultrasound Imaging, National-Regional Key Technology Engineering Laboratory for Medical Ultrasound, School of Biomedical Engineering, Shenzhen University Medical School, Shenzhen, 518060 China; 3 Kobilka Institute of Innovative Drug Discovery, School of Medicine, The Chinese University of Hong Kong, Shenzhen, Guangdong China; 4 Department of Pathology, The Second Affiliated Hospital, School of Medicine, The Chinese University of Hong Kong, Shenzhen & Longgang District People’s Hospital of Shenzhen, Shenzhen, 518172 China; 5 Institute of Digestive Disease, The Second Affiliated Hospital, School of Medicine, The Chinese University of Hong Kong, Shenzhen & Longgang District People’s Hospital of Shenzhen, Shenzhen, 518172 China; 6 Marshall Laboratory of Biomedical Engineering, Institute of Biological Therapy, Shenzhen University Medical School, Shenzhen University, Shenzhen, 518055 China; 7 The Chinese University of Hong Kong, Shenzhen Futian Biomedical Innovation R&D Center, Shenzhen, China

**Keywords:** Inflammatory bowel disease, Gene regulation

## Abstract

Intestinal fibrosis, as a late-stage complication of inflammatory bowel disease (IBD), leads to bowel obstruction and requires surgical intervention, significantly lowering the quality of life of affected patients. SAA3, a highly conserved member of the serum amyloid A (SAA) apolipoprotein family in mice, is synthesized primarily as an acute phase reactant in response to infection, inflammation and trauma. An increasing number of evidence suggests that SAA3 exerts a vital role in the fibrotic process, even though the underlying mechanisms are not yet fully comprehended. This study utilized dextran sulfate sodium (DSS) to establish an IBD mouse model and observed that the SAA3-deficient mice exhibited more severe intestinal fibrosis. Our results further indicated that SAA3 genetic disruption in fibroblasts enhanced cell activation to myofibroblasts through HSPB1/NF-κB/TGF-β1/Smads signaling cascade, exacerbating the pathological phenotype of intestinal fibrosis. Collectively, our results shed novel lights on regulating SAA3 in intestinal fibrosis and indicate the potential to develop therapeutic strategies for IBD patients.

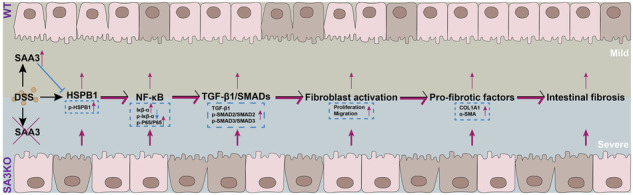

## Introduction

Inflammatory bowel disease (IBD), which includes Crohn’s disease (CD), microscopic colitis, ulcerative colitis (UC), and intermediate colitis, is the highly diverse group of conditions influencing a wide range of populations regardless of geographic or ethnic background [1, 2]. IBD has been reported worldwide, and its prevalence is high among western countries, showing an increasing trend among developing regions [3]. The pathogenesis of IBD is related to intestinal epithelial injury, mucosal inflammation, and dysbiosis, resulting in the dysregulation of intestinal mucosal barrier. This complex interplay involves gut microbial dysbiosis, genetic risk factors, abnormal host immune responses, and environmental factors [4, 5]. IBD can hardly be cured and is recurrent, which has significantly affected patient life quality. With the progress of the disease, most people undergoing IBD can develop more serious complications [6]. Intestinal fibrosis, usually caused by chronic inflammation, can be frequently observed in IBD without specific treatment. Particularly, more than 50% of CD patients and over 11% of UC patients with are affected by intestinal fibrosis [7]. Intestinal fibrosis, characterized by excessive tissue scarring on the intestinal mucosa due to chronic inflammation, is closely correlated with mesenchymal cell hyperplasia, extracellular matrix (ECM) deposition, and tissue disorganization [8, 9].

Recently, great achievements have been made in understanding molecular and cellular mechanisms related to intestinal fibrosis during IBD. Intestinal myofibroblasts and Th17 cells exert vital impacts on occurrence and development of intestinal fibrosis by interacting with profibrogenic pathways and cytokines to induce and maintain the fibrotic response [10, 11]. Moreover, genetic variants in cytokine genes, immunoregulatory proteins, and epigenetic factors have been implicated in intestinal fibrosis and IBD [12–15]. Studies have indicated that inhibition of fibroblast-expressed integrins activates latent TGFβ, therefore promoting a pro-fibrotic phenotype [16, 17]. Additionally, gut microbiota exerts a role in influencing fibrosis via the regulation of TGF-β1 expression and ECM protein deposition [18, 19]. Metabolic shifts from oxidative phosphorylation to glycolysis are also increasingly recognized as pathogenic processes in IBD-related fibrosis [20, 21].

The SAA proteins have several isoforms including SAA1 to SAA4, with SAA1 and SAA2 levels increasing significantly in acute-phase response, while SAA4 shows constitutive expression. Inducible expression of SAA occurs in the liver, and conversion of SAA to amyloid A (AA) results in amyloidosis [22, 23]. Although human SAA3 gene has been identified as the pseudogene, it is significantly upregulated in inflammatory tissues following LPS treatment in mouse models [24, 25]. SAA3 protein is present on colonic epithelium of mice with normal microbiota and exerts vital functions in opsonization, antimicrobial and chemotactic activities, and immunomodulation [26–28]. In addition, SAA3 also has been implicated in the development of atherosclerosis through increasing cholesterol efflux capacity in macrophages, inducing pathogenic Th17 cells, and contributing to kidney injury in the mouse model of type I diabetes [29, 30]. Peter Verstraelen et al. has reported SAA3 as a key mediator of gastrointestinal vulnerability to bacterial-derived amyloids, showing the potential of dual leucine zipper kinase inhibition to dampen enteric pathology [31]. A recent study suggests that SAA3 is vital for fibrosis occurrence, serving as an early indicator of renal fibrosis [32]. The use of in vivo bioluminescence imaging of the Saa3/C/EBPβ promoter proved to be a sensitive tool for detecting and visualizing of tubulointerstitial fibrosis, which was related to TNF-α and collagen I expression levels in the injured kidney.

This study established an SAA3-deficient IBD mouse model and observed severe intestinal fibrosis in genetically engineered mice following the induction of IBD. Using CRISPR-mediated disruption of SAA3 in NIH3T3 fibroblasts, we demonstrated that DSS administration significantly increased SAA3 and HSPB1 expression. Elevated SAA3 partially inhibited HSPB1 and p-HSPB1 expression, which in turn attenuated the NF-κB pathway. Deletion of SAA3 abolished the inhibitory effect on HSPB1 and obviously elevated the expression of HSPB1 and p-HSPB1, causing increased NF-κB signal flux. In addition, enhanced NF-κB signaling leads to increased levels of TGF-β1 and its downstream mediators, Smad proteins. Therefore, the increased TGF-β1/Smad signaling pathway accelerates fibroblast activation, thereby facilitating the progression of intestinal fibrosis. Based on these findings, SAA3 plays a vital role in regulating fibroblast behavior and the development of intestinal fibrosis, providing valuable insights into potential therapeutic strategies for treating fibrotic conditions.

## Results

### SAA3 gene deficiency significantly exacerbates IBD pathological phenotypes

**Fig. 1:**
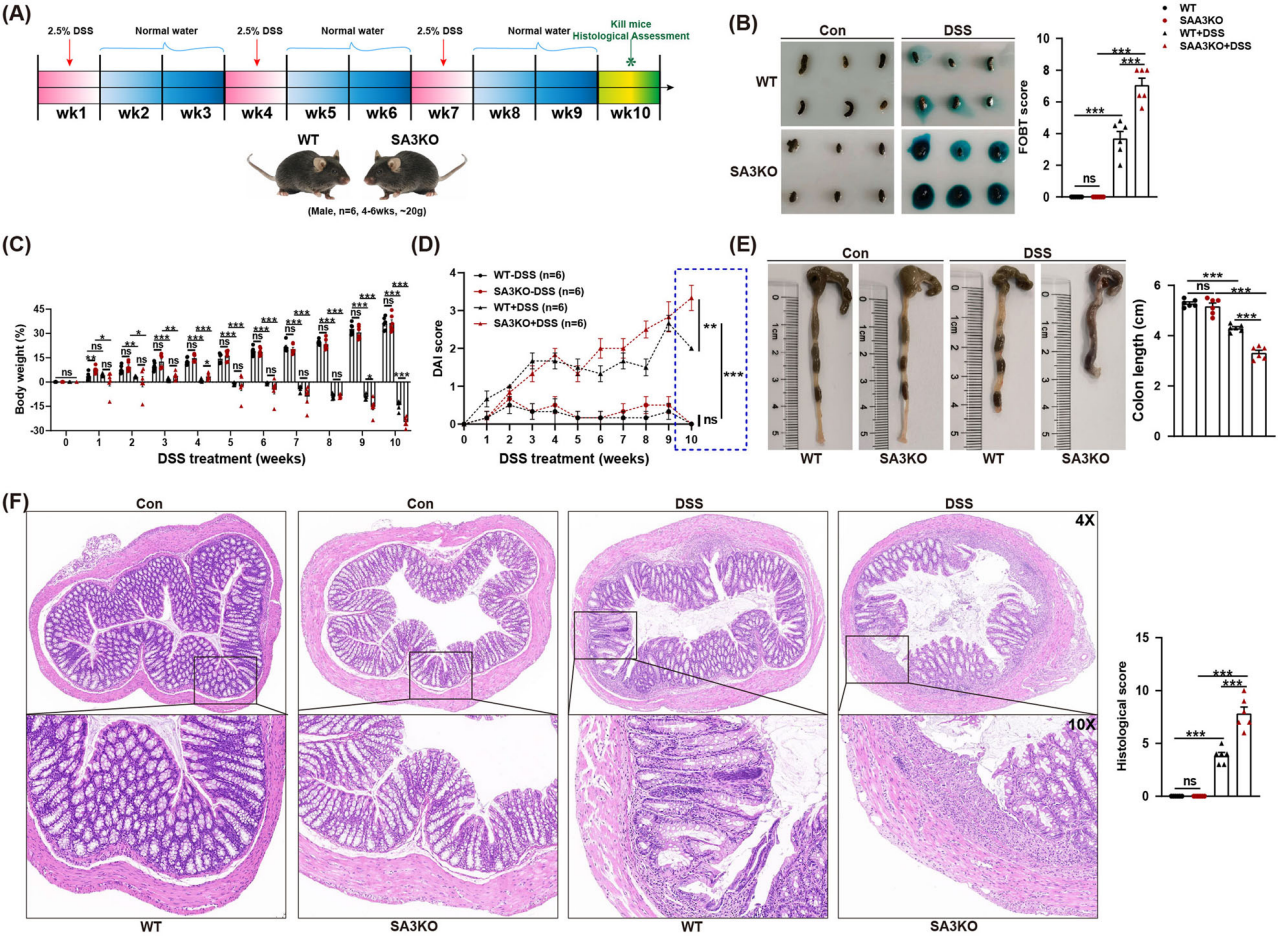
The absence of the SAA3 gene significantly exacerbates IBD phenotypes. **A** Schematic representation of DSS-induced IBD mouse model. **B** Feces collected for occult blood testing and scoring. *N* = 6. ns, not significant; ***P < 0.001. **C** Detection of changes in mouse body weight during IBD modeling. *N* = 6. ns, not significant; *P < 0.05; **P < 0.01; ***P < 0.001. **D** The DAI score of the IBD mouse model. *N* = 6. ns, not significant; **P < 0.01; ***P < 0.001. **E** Ten weeks later, the mice were sacrificed and colonic tissues were collected to measure their length. *N* = 6. ns, not significant; **P < 0.01; ***P < 0.001. **F** Representative images of H&E-stained colon sections from the control and DSS-treated mice after ten weeks. Histological scores of colon pathology are shown in the right panel. *N* = 6. Scale bars: 50 and 20 μm in the upper and lower panels, respectively. ns, not significant; *P < 0.05; ***P < 0.001.

In this study, IBD mice were induced with 2.5% DSS and sacrificed on week 10 for phenotypic analysis (Fig. 1A). Fecal occult blood test results indicated that SA3KO mice had severely bloody stools when compared with WT mice after DSS administration (Fig. 1B). DSS-treated mice exhibited progressive weight loss compared to controls, with SA3KO mice representing accelerated weight loss from week 9 (Fig. 1C). DAI score of the SA3KO group was significantly higher than that of the WT group 10 weeks after DSS treatment (Fig. 1D). After DSS treatment, SA3KO mice had significantly shorter colon lengths than their WT littermates (Fig. 1E). Histological analysis of H&E-stained colon tissue indicated more severe epithelial damage in SA3KO mice, with a severe loss of crypts after DSS administration (Fig. 1F). The obtained findings show that loss of SAA3 results in a more severe pathological progression of IBD.

### Genetic deletion of SAA3 significantly accelerates the progression of IBD intestinal fibrosis

**Fig. 2:**
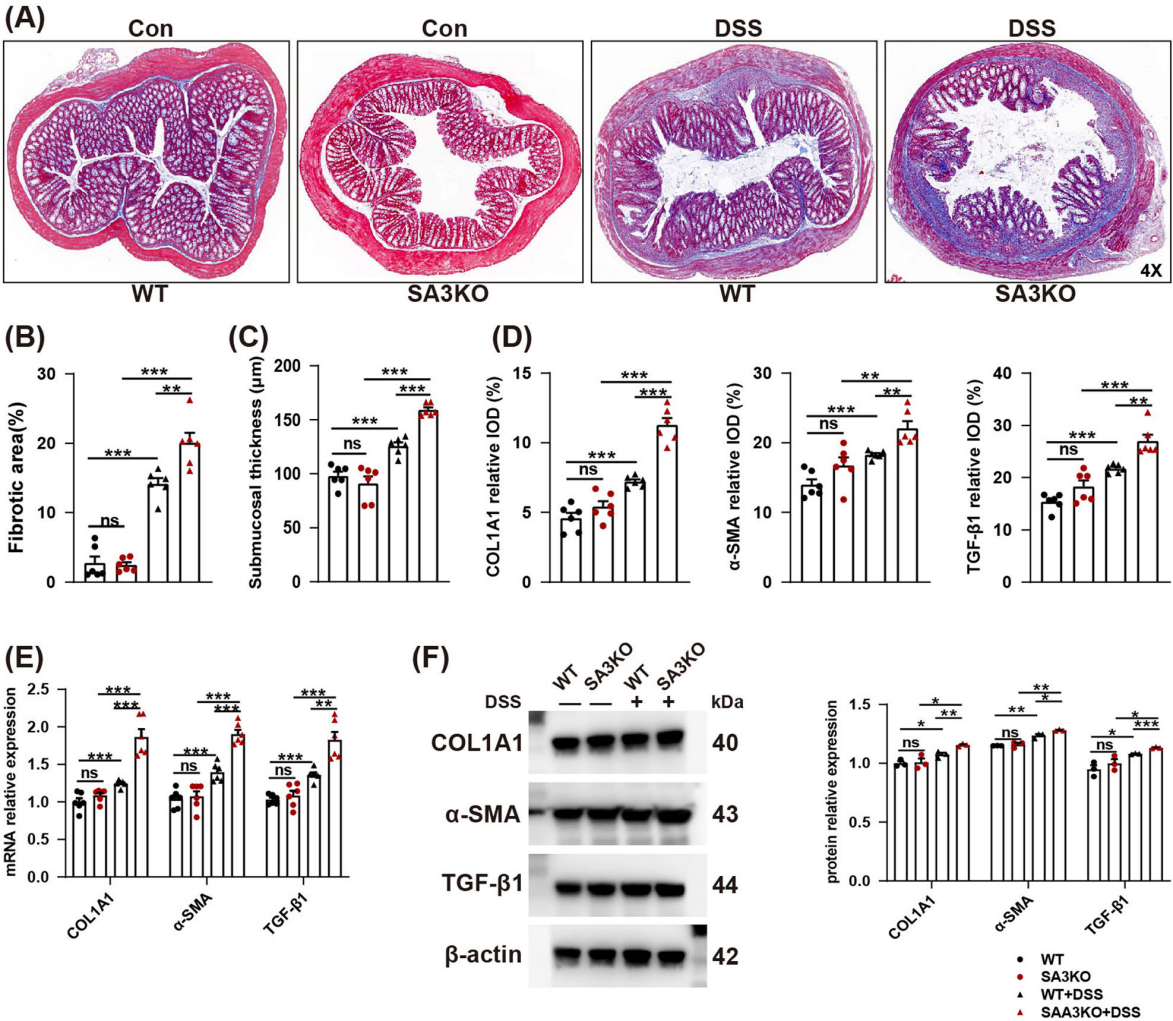
SA3KO mice exhibited severe intestinal fibrosis after DSS treatment. **A**, **B** Masson’s trichrome staining was performed to evaluate the severity of intestinal fibrosis in each group. *N* = 6. scale bar = 50 μm. ns, not significant; **P < 0.01; ***P < 0.001. **C** Submucosal thickness is measured as an indicator of fibrosis. *N* = 6. ns, not significant; ***P < 0.001. **D** Quantitative analysis of fibrotic gene expression was performed on IHC-stained colon sections from DSS-treated mice relative to WT mice. *N* = 6. ns, not significant; **P < 0.01; ***P < 0.001. **E** RT-qPCR was used to detect the mRNA expression of COL1A1, α-SMA, and TGF-β1 in each group. *N* = 6. ns, not significant; **P < 0.01; ***P < 0.001. **F** WB results for COL1A1, α-SMA, and TGF-β1 expression in WT and SA3KO mice. *N* = 3, representative biological replicates; ns, not significant; *P < 0.05; **P < 0.01; ***P < 0.001.

Then, the pathological process of intestinal fibrosis was evaluated in SA3KO and WT mice after DSS treatment. The results of Masson’s trichrome staining and changes in submucosal thickness demonstrated that intestinal fibrosis was more severe in SA3KO mice relative to that in WT littermates after DSS treatment (Fig. 2A–C). Subsequently, IHC, qRT-PCR, and Western blot (WB) results demonstrated that SA3KO mice showed significantly increased fibrosis-related gene transcript and protein levels, including type I collagen α1 (COL1A1), transforming growth factor beta1 (TGF-β1), and α-smooth muscle actin (α-SMA) in relative to WT mice following DSS treatment (Fig. 2D–F and Supplementary Fig. S1). Collectively, SAA3 deficiency leads to more severe intestinal fibrosis.

### SAA3 deficiency enhanced fibroblast activation after DSS treatment

**Fig. 3:**
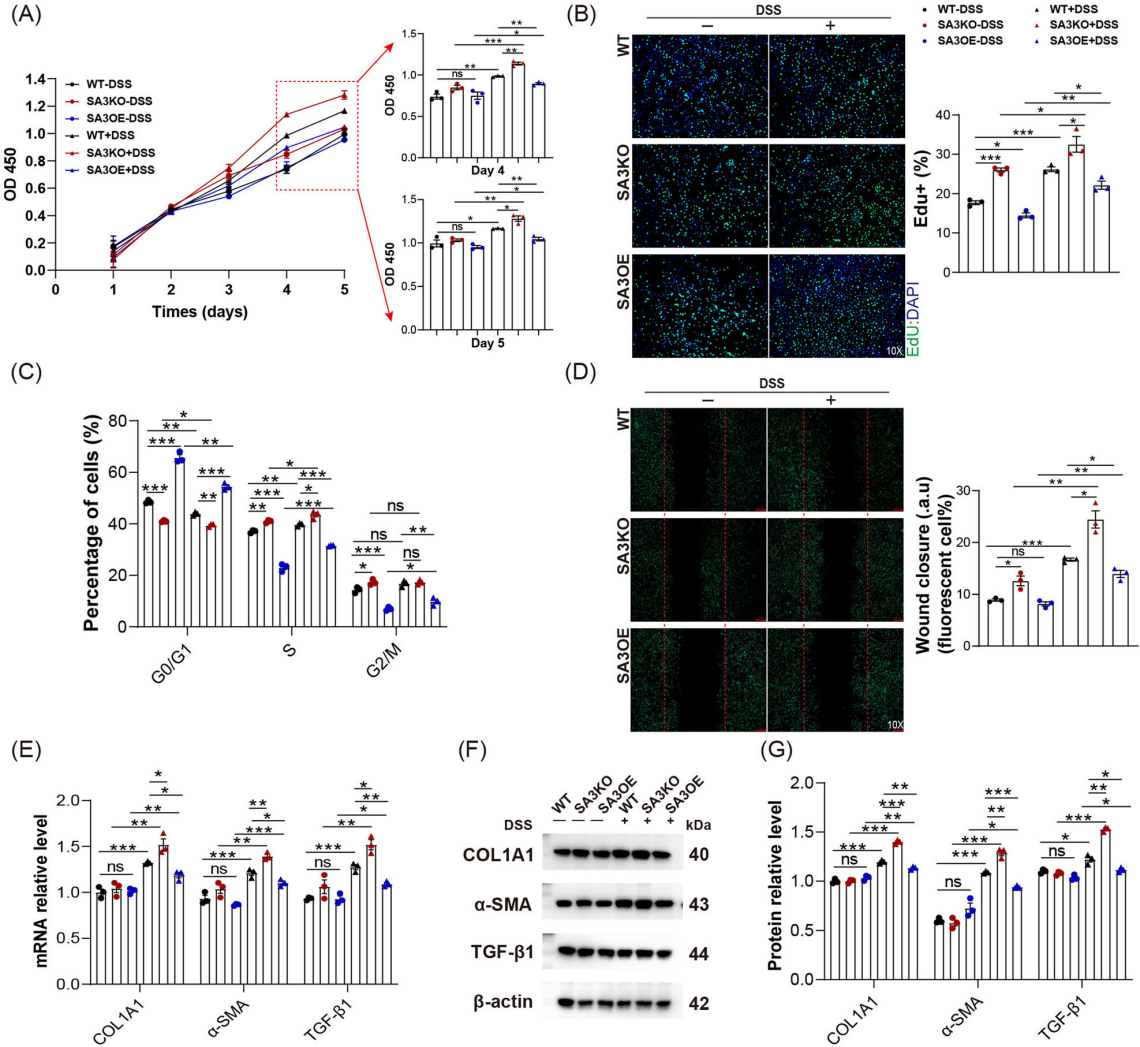
SAA3 deficiency increases fibroblast activation. **A** The CCK8 assay was applied to detect cell viability. *N* = 3, biological replicates; ns, not significant; *P < 0.05, **P < 0.01, ***P < 0.001. **B**, **C** EdU staining and cell cycle analysis were performed to evaluate the fibroblast proliferation in each group. *N* = 3, biological replicates; scale bar = 20 μm; ns, not significant; *P < 0.05; **P < 0.01; ***P < 0.001. **D** Fibroblast migration ability was determined using a wound-healing assay for each group. *N* = 3, biological replicates; scale bar = 20 μm; ns, not significant; *P < 0.05; **P < 0.01; ***P < 0.001. **E**–**G** COL1A1, α-SMA, and TGF-β1 mRNA and protein expression were analyzed for each group. *N* = 3, biological replicates; ns, not significant; *P < 0.05; **P < 0.01; ***P < 0.001.

To clarify how SAA3 was involved in intestinal fibrosis pathogenesis during IBD, CRISPR/Cas9 and lentiviral technologies were used to establish SAA3 gene knockout (SA3KO) and overexpression (SA3OE) cell lines using NIH3T3 mouse fibroblasts. At first, the effect of SAA3 was investigated on fibroblast activation. In CCK8 assays, a progressive increase of cell viability could be observed in both groups following DSS treatment, and a notable increase in cell viability was detected in SA3KO cells from day 4 compared to WT cells (Fig. 3A). Moreover, as presented in Fig. 3B, C and Supplementary Fig. S2B–D, after DSS treatment, SA3KO cells exhibited significantly increased cell proliferation ability and accelerated cell cycle progression compared to WT cells, while apoptosis was unaffected, as indicated by EdU staining, cell cycle analysis, and apoptosis analysis, respectively. Consistent with the above-mentioned findings, wound healing assays showed that SA3KO cells exhibited enhanced migration ability compared to WT cells after DSS treatment (Fig. 3D). Moreover, the transcript and protein expression of myofibroblast markers (COL1A1, α-SMA, and TGF-β1) significantly increased in SA3KO cells compared to WT cells after DSS treatment (Fig. 3E–G). Significantly, all of these observed effects were reversible by overexpression of the SAA3 gene. The obtained data strongly suggest that disruption of SAA3 improves fibroblast activation.

### SAA3 deficiency promotes NF-κB activation

**Fig. 4:**
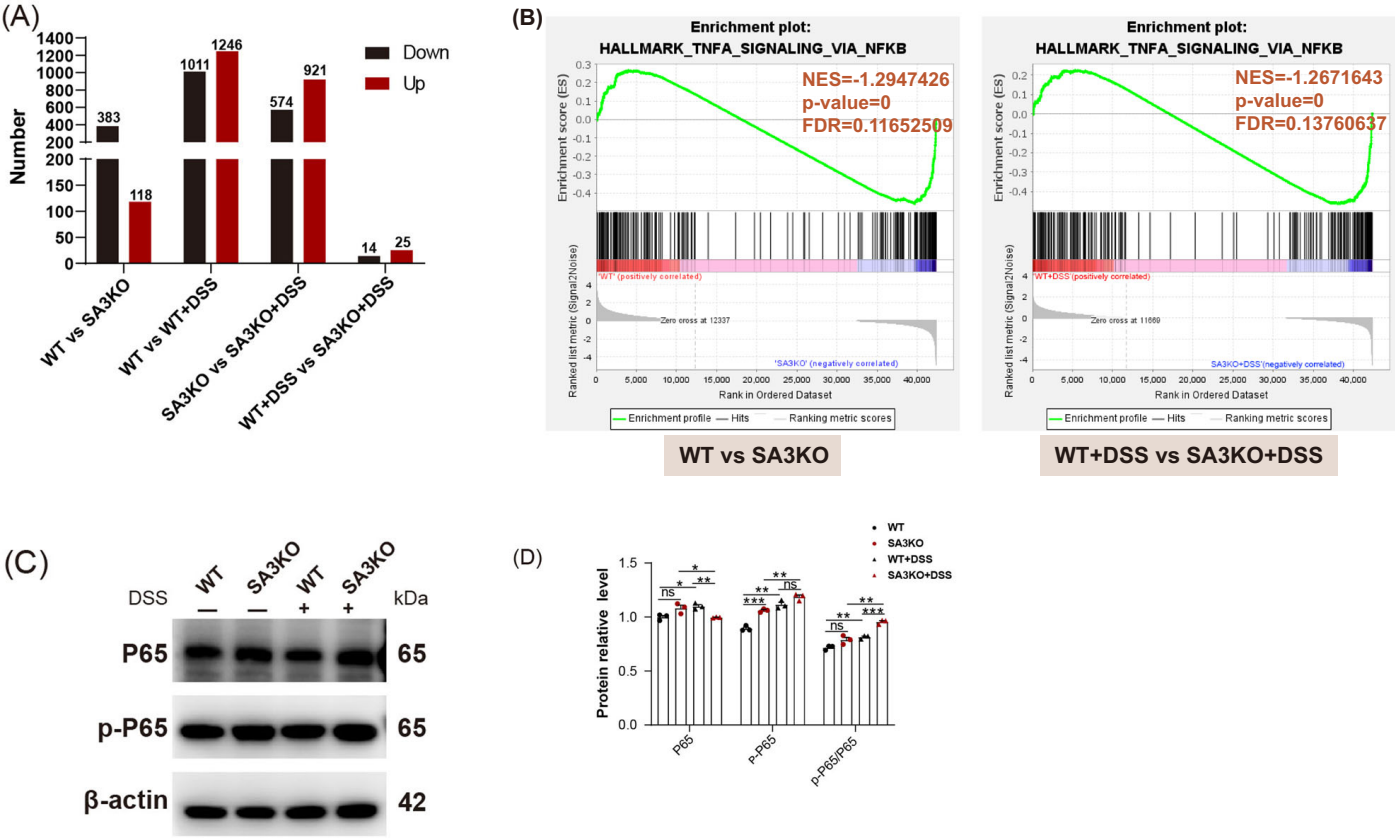
SAA3 deficiency enhances the activation of the NF-κB pathway. **A** Significantly differentially expressed genes (DEGs) between the groups were analyzed. **B** The NF-κB pathway was enriched in WT and SA3KO mice according to GSEA. **C**, **D** NF-κB pathway activation was verified through WB analysis. *N* = 3, representative biological replicates; ns, not significant; *P < 0.05; **P < 0.01; ***P < 0.001.

To investigate how SAA3 affected IBD intestinal fibrosis, three colon tissues from each group were prepared for transcriptome analysis. Comparative analysis indicated that in the SA3KO group, 383 genes exhibited down-regulation, while 118 showed up-regulation in SA3KO group relative to WT littermates. Clearly, after DSS treatment, only 14 genes showed down-regulation, while 25 revealed up-regulation in SA3KO group (Fig. 4A). According to subsequent GSEA, DSS treatment activated NF-κB pathway, and SAA3 gene deficiency promoted activation of the NF-κB pathway. (Fig. 4B). Based on further validation of these findings at the protein level, the ratio of P65 to its phosphorylated form (p-P65), a well-established indicator of NF-κB activity, was significantly upregulated after DSS treatment. Particularly, the P65/p-P65 ratio was much higher in SA3KO mice compared to the WT group, indicating a stronger signal flux (Fig. 4C, D). These results strongly suggest that SAA3 deficiency improves the activation of the NF-κB pathway.

### Loss of SAA3 enhances the NF-κB pathway through HSPB1

**Fig. 5:**
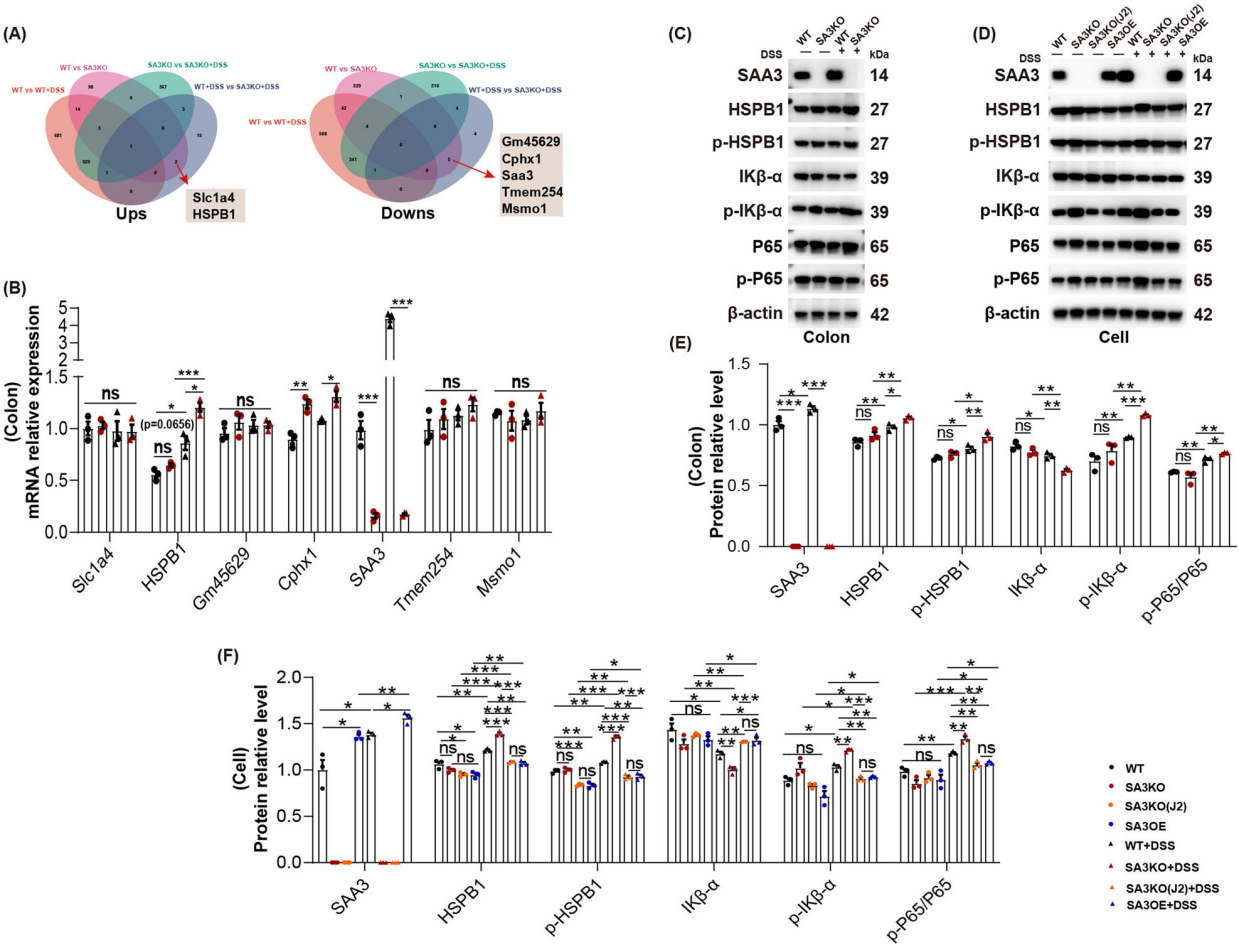
SAA3 deficiency promotes activation of the NF-κB pathway through HSPB1. **A** Construction of a Venn diagram of DEGs between the groups. **B** Gene expression changes affecting the colonic tissues of WT and SA3KO mice were verified by qRT-PCR. *N* = 3, representative biological replicates; ns, not significant; *P < 0.05; **P < 0.01; ***P < 0.001. **C**–**F** WB analysis of crosstalk between HSPB1 and the NF-κB pathway. *N* = 3, representative biological replicates; ns, not significant; *P < 0.05; **P < 0.01; ***P < 0.001.

In Venn diagram clarifying DEGs, the SAA3 KO groups evidently showed two upregulated genes (Slc1a4 and HSPB1) and five downregulated genes (Gm45629, Cphx1, SAA3, Tmem254 and Msmo1) when compared with the WT littermates, regardless of DSS treatment (Fig. 5A). Subsequently validated at the transcriptional level, RT-qPCR results suggested that HSPB1 expression increased after SAA3 deletion, while no significant difference was observed (*P* = 0.0656). Notably, after DSS treatment, HSPB1 expression of SA3KO group significantly increased relative to WT group (Fig. 5B). It has been previously suggested that the HSPB1 and its phosphorylated form (p-HSPB1)-mediated IKBα-NF-κB signaling axis promotes pulmonary fibrosis progression [33]. Therefore, this study further investigated whether HSPB1 was related to IBD intestinal fibrosis. Based on WB results, DSS treatment significantly up-regulated SAA3 and HSPB1 as well as its phosphorylated form. In particular, the increased SAA3 expression partially suppressed HSPB1 expression. Moreover, the SAA3 gene deletion abolished the inhibitory effect and significantly up-regulated HSPB1 and p-HSPB1, therefore enhancing NF-κB signal flux, which could be reversed by SAA3 gene overexpression. Furthermore, we found that inhibition of HSPB1 and p-HSPB1 expression using J2, a small molecule covalently binding to HSPB1 and inhibiting its oligomerization activity [33],could reduce NF-κB signaling, consistent with the effects observed with SAA3 gene overexpression (Fig. 5C–F). These findings indicate that SAA3 deficiency enhances NF-κB signaling by relieving the inhibitory effect on HSPB1 and upregulating the expression of HSPB1 and its phosphorylated form, which can therefore enhance the NF-κB signaling pathway after DSS treatment.

### Loss of SAA3 increases TGF-β1/Smads via the NF-κB pathway

**Fig. 6:**
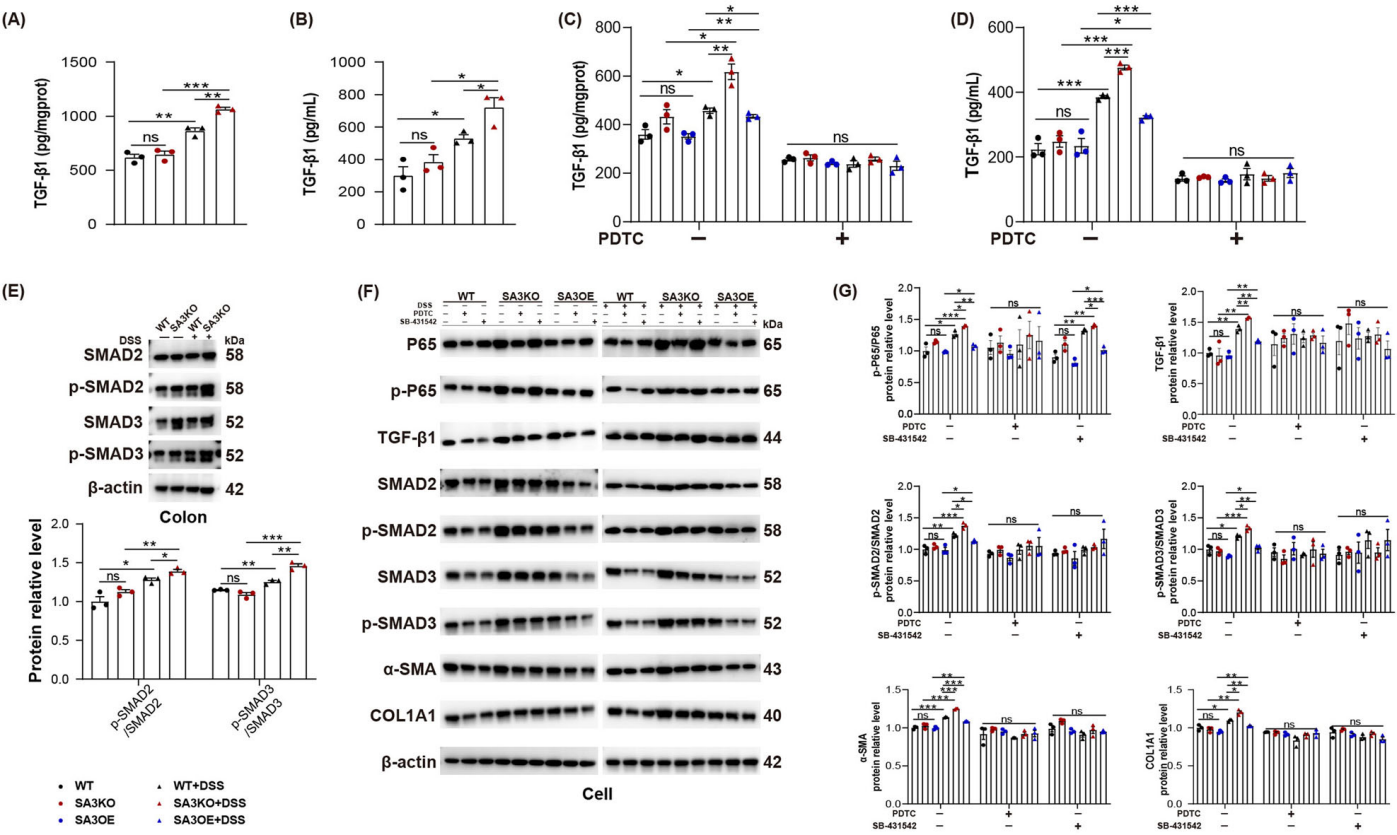
SAA3 deficiency increases TGF-β1/ Smad levels through the NF-κB pathway. **A**, **B** TGF-β1 expression was measured in colonic tissue and serum of SA3KO and WT mice with or without DSS. *N* = 3, representative biological replicates; ns, not significant; *P < 0.05; **P < 0.01; ***P < 0.001. **C**, **D** TGF-β1 levels were detected in the cell lysates and culture supernatants of each group treated with or without PDTC. *N* = 3, biological replicates; ns, not significant; *P < 0.05; **P < 0.01; ***P < 0.001. **E**–**G** Changes in NF-κB/ TGF-β1/ Smad molecules were observed in WT, SA3KO, and SA3OE fibroblasts by WB analysis. *N* = 3, representative biological replicates; ns, not significant; *P < 0.05; **P < 0.01; ***P < 0.001.

The interplay of TGF-β1 with NF-κB pathway is implicated in fibrosis-related diseases [34, 35]. Our investigation revealed the significantly increased TGF-β1 in serum and colon tissue in SA3KO mice relative to WT mice following DSS treatment (Fig. 6A, B). Similarly, there was a significant increase in the secretion of TGF-β1 into the medium and its expression in the cell lysate in SA3KO cells after DSS treatment, whereas these impacts were reversible in SA3OE cells. Notably, PDTC, the NF-κB pathway inhibitor, significantly decreased TGF-β1 expression. SB-431542, as a selective inhibitor of the TGF-β type I receptor, did not influence the ratio of p-P65/P65, suggesting that TGF-β1 is the downstream signal of NF-κB (Fig. 6C, D, F, G).

TGF-β1 is identified to be a vital regulator for fibrosis, primarily inducing scarring through the activation of its downstream Smad pathway [36]. Subsequent WB results suggested that SAA3 deficiency increases Smad signaling, as demonstrated by upregulation of the ratio of phosphorylated SMAD2/3 to SMAD2/3 (Fig. 6E). Both PDTC and SB-431542 significantly suppressed the expression of Smads and fibrotic genes in SA3KO fibroblasts, conforming to the effects observed upon SAA3 gene overexpression (Fig. 6G and Supplementary Fig. S3). SAA3 deficiency increases TGF-β1/Smads via the NF-κB pathway.

## Discussion

This study indicated that the SAA3-deficient mice developed more severe intestinal fibrosis than the WT mice in the IBD mouse model. Mechanistically, in fibroblasts, DSS administration significantly increased SAA3 and HSPB1. Elevated SAA3 partially inhibited HSPB1 and p-HSPB1 expression, which in turn attenuated the NF-κB pathway. Deletion of SAA3 abolished the inhibitory effect on HSPB1 and obviously elevated the expression of HSPB1 and p-HSPB1, causing increased NF-κB signal flux. This further enhanced the expression of TGF-β1 and the activation of the downstream Smads pathway, as well as stimulated the transformation of fibroblasts into myofibroblasts, ultimately exacerbating fibrosis.

A growing body of studies have emphasized the correlation of SAA with fibrosis-related diseases. Studies consistently indicate increased levels of SAA among idiopathic pulmonary fibrosis (IPF) patients relative to healthy individuals [37]. Moreover, people with IPF and fibrotic sarcoidosis exhibit higher levels of SAA than those with IPF alone [38]. Particularly, SAA has been implicated in promoting M2b-like macrophage polarization during liver inflammation, thus attenuating fibrogenesis [39]. In addition, SAA has been associated with early renal dysfunction by activating IFN-γ-iNOS-p38 MAPK pathway, causing increased renal tissue fibrosis [40]. This study has explored whether SAA can be the biomarker for assessing drug efficacy and inflammatory responses in cystic fibrosis, as well as a marker for lung infection in these patients [41, 42]. Research suggests that cancer stem cell-derived SAA exerts a vital role in promoting tumor fibrosis and treatment resistance by influencing type 2 immune polarization and cancer stemness transformation through the P2X7 receptor [43].

Our findings indicated that SAA3 gene deficiency led to severe intestinal fibrosis in the IBD mouse model. Human SAA3 (hSAA3) shows different characteristics compared with other serum amyloid A isoforms, SAA1, SAA2 and SAA4. It has long been considered as a pseudogene when compared with the abundant expression of the other isoforms [44]. Nevertheless, mouse SAA3 (mSAA3) expression is known to be upregulated extrahepatically in inflammatory responses and serves as an endogenous ligand for the toll-like receptor 4/MD-2 complex [45]. In a recent study, hSAA3 was shown to be transcribed into hSAA2-SAA3 fusion transcripts in different human cells. hSAA2 exon 3 was correlated with hSAA3 exon 1 or exon 2 in a fusion transcript, which is located approximately130kb downstream hSAA2 exon 3 in the genome, suggesting its generation through alternative splicing. It is the first time that researchers were capable of detecting and isolating hSAA3 protein using an immunoprecipitation-enzyme immunoassay system with monoclonal and polyclonal antibodies recognizing unique amino acid sequence of hSAA3 [46]. Although it is very difficult to detect hSAA3 expression and its role in human intestinal inflammation and/or fibrosis associated with IBD, investigating its homologous genes in mice may provide insights into conserved mechanisms during the processes of inflammation and fibrosis, which may be valuable in developing therapeutic strategies for IBD.

It is important to consider that while the direct translation of findings from mouse models to humans can be complex due to genetic and physiological differences, certain inflammatory pathways and fibrotic processes may be conserved between species. The upregulation of mSAA3 during inflammatory responses may provide a model for studying the inflammatory milieu of human IBD. Understanding how mSAA3 contributes to fibrosis could guide future research exploring the mechanisms of hSAA3 in human intestinal inflammation, despite the challenges in detecting hSAA3 owing to its low expression levels. Investigating the mechanisms underlying mSAA3-induced fibrosis may reveal similar pathways in humans, particularly how these pathways contribute to the pathogenesis of IBD-related tissue remodeling. By focusing on the homologous functions and regulatory pathways of SAA3 in both models, researchers may identify therapeutic targets that can be used in human clinical contexts. In conclusion, while mSAA3 and hSAA3 exhibit notable differences, understanding the role of SAA3 in mouse models of IBD is a critical step toward uncovering analogous processes in human disease. Continued research on the expression and function of hSAA3 in human IBD may ultimately lead to the development of effective therapeutic strategies for patients suffering from this complex condition.

In this study, HSPB1 and its phosphorylated form were significantly up-regulated during IBD intestinal fibrosis. HSPB1, as a member of small HSP family, shows up-regulation in response to stress. Serving as the molecular chaperone sensor, HSPB1 undergoes dynamic phosphorylation and oligomerization changes, which enables cells to effectively adapt to physiological changes and mount a protective response to injury [47, 48]. Recently, HSPB1 has been demonstrated with multiple roles in fibrotic process. In PF, HSPB1 regulates TGF-β-mediated lung fibroblast differentiation via the Smad3 and ERK pathways and contributes to radiation-induced lung fibrosis via the activation of the IkBα-NFκB signaling [33, 49]. Besides, the up-regulated miR-15b expression transcriptionally inhibits Smurf2, which hinders the degradation of pHSP27, thereby exacerbating the progression of PF [50]. In liver fibrosis, HSPB1 activates JAK2/STAT3 and TGF-β1/Smad pathways [51], while it regulates cell proliferation and directly interacts with JAK2/STAT5 in myelofibrosis [52]. Additionally, HSPB1 exerts a role in Crohn’s disease by mediating TNF-α-stimulated myofibroblast migration and contributes to renal tubulointerstitial fibrosis by modulating E-cadherin expression through Snail downregulation [53, 54]. Moreover, this study further elucidates that HSPB1 is related to IBD intestinal fibrosis and also provides a valuable target for treatment.

Previous studies have indicated the involvement of both NF-κB and TGF-β1 in liver fibrosis [55]. In cardiac fibrosis, the loss of the MYBPC3 gene activates NF-κB pathway, resulting in increased TGF-β1 production and increased aerobic glycolysis, which facilitate the occurrence of fibrosis [34]. While NF-κB and TGF-β signaling pathways exert vital roles in various fibrotic processes, the specific mechanism linking NF-κB and TGF-β is unclear. The study demonstrated DSS-induced NF-κB pathway activation in an IBD mouse model, and SA3KO mice exhibited more enhanced NF-κB flux than WT mice after DSS treatment. In fibroblasts, DSS treatment significantly increased SAA3 and HSPB1 expression, and the increased SAA3 could partially inhibit HSPB1 expression, and reduce NF-κB signal flux. Using the NF-κB signaling pathway inhibitor PDTC and SB-431542, a selective inhibitor of TGF-β1, we confirmed that NF-κB promoted TGF-β1 expression and activation of the Smads signaling pathway, driving the transition from fibroblasts to myofibroblasts and exacerbating intestinal fibrosis in IBD. These findings not only elucidate the mechanism of the interaction between NF-κB and TGF-β1, but also provide novel ideas for investigating therapeutic strategies for intestinal fibrosis.

In conclusion, our study demonstrated that SAA3 deficiency in fibroblasts promotes the transition to myofibroblasts through HSPB1/NF-κB/TGF-β1/Smads signaling pathway, exacerbating intestinal fibrosis in IBD. Moreover, the results shed novel lights on regulating SAA3 in intestinal fibrosis and offer promising avenues for developing targeted treatments for people with IBD.

## Materials and methods

### Ethical approval

Our animal experimental protocols were approved by Animal Welfare and Research Ethics Committee, the Chinese University of Hong Kong, Shenzhen (CUHKSZ-AE2021006), and strictly adhered to the principles outlined in Guide for the Care and Use of Laboratory Animals. All surgical procedures were carried out under anesthesia and all measures were taken to minimize pain and distress to the animals.

### Animals

SAA3 KO mice were obtained from the Knockout Mice Project Repository (Davis, CA) and bred under the C57BL/6 genetic background. The 6-8-week-old male mice (weighing ~20 g) were maintained in the specific pathogen-free environment, and allowed to drink water and eat food freely at Chinese University of Hong Kong, Shenzhen Laboratory Animal Center. Weight- and sex-matched SAA3 KO and wild-type (WT) littermates were applied in all the experiments. Animal experimental protocols were approved by Animal Care and Research Ethics Committee of the Chinese University of Hong Kong, Shenzhen (CUHKSZ-AE2021006).

### DSS-induced IBD model and phenotype evaluation

The 2.5% (w/v) DSS (TdB Labs AB, DB001-4, Sweden) was added to induce IBD in mice (each group *n* = 6) that were provided free drinking water for 1 week at 2-week intervals for a cycle of three cycles. After adding DSS to the drinking water, mouse body weight and fecal occult blood were monitored weekly. Ten weeks after DSS administration, SAA3 WT and KO mice were sacrificed to measure the colon length and histology.

For fecal occult blood test (FOBT), the feces of each group of mice were collected and placed on a white plate. Meanwhile, 10 g/L benzidine solution was added, followed by 3% hydrogen peroxide solution, with the time and degree of staining being recorded. For mice that died and did not reach the endpoint during the study, their fecal samples were not included. Fecal occult blood scores were rated below, 0= non-staining within 2 min, 2= staining within 1 min, 5= immediate taining to blue, 8= immediate staining to dark blue. A proportional calculation could be employed to calculate specific scores based on the time of color change relative to the described stages.

Regarding the Disease Activity Index (DAI), weight loss, stool consistency and blood stool were scored in accordance with a standard scoring system [56], which was calculated as the sum of the above three aspects for each mouse.

For Masson’s trichrome and H&E staining, fresh colons were immersed in 4% PFA, followed by dehydration, paraffin embedding, and sectioning into 5-μm sections. Then, two sections of uniform thickness and intact histological structure were selected. One respective section was used for Masson’s trichrome and H&E staining by standard techniques. As previously described, the severity of tissue injury and inflammation was explored in a blinded fashion [57]. A proportional calculation could be employed to calculate specific scores based on inflammation and tissue injury severity relative to the stages described. regarding fibrosis analysis, total tissue area, fibrotic area (blue), and submucosal thickness were calculated using ImageJ software (NIH, Bethesda, MD, USA).

Regarding immunohistochemistry (IHC) analysis, paraffin-embedded colon sections were deparaffinized. In addition, antigen retrieval was performed based on standard techniques. Subsequently, sections were rinsed with PBS, blocked with 5% BSA, incubated with antibodies, stained with DAB, and mounted with neutral resin. ImageJ software was used to explore protein abundance in each section. Supplementary Table 1 presents primary and secondary antibodies.

### Quantitative reverse transcription PCR (qRT-PCR)

With the purpose of determining relative gene mRNA expression, FastPure Cell/Tissue Total RNA Isolation Kit (Vazyme, RC112, China) was employed for extracting total RNA following the specific protocols. Subsequently, RNA (1 μg) was prepared into cDNA through reverse transcription with HiScript IV RT SuperMix for qPCR Kit (with gDNA wiper) (Vazyme, R423, China) in line with manufacturer’s instructions. qRT-PCR was carried out with Taq Pro Universal SYBR qPCR Master Mix Kit (Vazyme, Q712, China). Meanwhile, QuantStudio 3 Real-Time PCR System (Thermo Fisher Scientific, ABI, iQ3) was used for analyzing amplification plots and fluorescence intensity. The 2^-ΔΔCT^ method was applied to normalize data to β-actin expression. Error bars indicate standard error of mean (SEM) for samples performed in biological triplicate. Primers applied in the qRT-PCR are displayed in Supplementary Table 2.

### Western blot (WB)

Samples were first lysed in RIPA lysis buffer (Beyotime, P10013B, China) containing protein inhibitors. Later, the samples were centrifuged to collect supernatants containing total protein. Next, protein aliquots (40 μg) were subjected to SDS-PAGE for separation prior to transfer onto PVDF membranes (Epizyme, WJ002, China). Then, they were blocked with the blocking buffer (Epizyme, PS108P, China) for 2 h and incubated with antibodies. SuperFemto ECL chemiluminescence kit (Vazyme, E423, China) was used for visualizing protein bands. Supplementary Table 1 presents primary and secondary antibodies.

### Cell culture

NIH3T3 cells offered by Procell Life Science & Technology Co. (Wuhan, China) were cultured in DMEM (TransGen Biotech, FI101-01, China) supplemented with 10% FBS (Vazyme, F101-01, China), penicillin and streptomycin under 37 °C with 5% CO2. Cells were free from mycoplasma infection.

### Establishment of SAA3 gene knockout (SA3KO) cell lines

The CRISPR/Cas9 system targeting the mouse SAA3 gene was constructed based on the methods described in a previous study [34]. The sgRNA oligonucleotides were synthesized (GENEWIZ, China), annealed and then cloned into the vector (Addgene #42230). Subsequently, NIH 3T3 cells were electrotransfected with 30 μg of plasmids, and plated in 10 cm dishes. Single cell colonies were collected and cultivated in the 24-well plates. 10% of the cells from each plate were lysed for detecting positive clones through PCR. The forward primer was 5′-GGGTTTCAGCTGGCATGATTGG-3′, and the reverse primer was 5′-CCCGTGGAAAGACTGCTGTA-3′. The PCR conditions were 94 °C for 5 min; 94 °C for 30 s, 60 °C for 1 min, and 72 °C for 40 s for 35 cycles; 72 °C for 5 min; as well as a hold at 16 °C. Mutations were detected in PCR products through sequencing (Supplementary Fig. S2A). Finally, positive cell colonies were expanded and cryopreserved.

### Establishment of SAA3 gene overexpression (SA3OE) cell lines

At first, the CDS sequence of the SAA3 gene was amplified and inserted into a lentivirus overexpression vector. Then, the lentivirus was packaged and virus titer assayed following the specific protocols. Subsequently, the lentivirus was introduced into NIH 3T3 cells and puromycin was added to the medium for a screening period of 7 days. With cells reaching 90% confluence, 10% were collected in each plate and genotyped by qRT-PCR, followed by expansion and cryopreservation of positive cell colonies. Supplementary Table 2 displays the used primers for qRT-PCR.

### Cell viability assay

NIH 3T3 cells were inoculated into 96-well plates in equal numbers, prior to DSS treatment at different concentrations (0 or 0.1%). After 1–5 days of incubation, CCK-8 cell counting kit (Vazyme, A311, China) was used to assess cell viability in line with the specific protocols. CCK-8 solution (10 μL) was introduced into every well for 2 h of incubation. Next, absorbance values were identified with an EnVision 2015 Multimode Plate Reader (PerkinElmer, USA) at 450 nm.

### EdU staining

BeyoClick™ EdU-488 kit (Beyotime, C0071, China) was used to assess cell proliferation ability in line with the specific protocols. In brief, cells (5 × 10^5^/well) were inoculated into 24-well plates, followed by 2 h of incubation using 10 μM EdU. After 10-min fixation in 4% PFA, cells were subjected to 15 min of permeabilization in PBS/0.3% Triton X-100, rinsing three times by PBS, and staining using DAPI for 10 min. Samples were examined by fluorescence microscopy. In addition, ImageJ was used for quantifying EdU-positive cells.

### Cell cycle profile

Cell cycle was assessed with a cell cycle analysis kit (Beyotime, C1052, China) based on the instructions of the manufacturer. Cells were harvested, washed twice with PBS, fixed in 70% cold ethanol overnight at 4 °C and subsequently stained with propidium iodide (PI) for 30 min in the dark at 37 °C. Then, the distribution of cells in different phases of the cell cycle was explored through measuring DNA content with a CytoFLEX flow cytometer (Beckman Coulter, Brea, CA). With the use of FlowJo software, the populations of cells in G1, S, and G2/M phases were identified.

### Cell apoptosis assay

Annexin V-FITC apoptosis detection kit (Beyotime, C1062, China) was adopted for assessing cell apoptosis following the specific protocols. Briefly, by collecting NIH3T3 cells, Annexin V-FITC and PI were added in binding buffer to incubate cells under 4 °C in dark for 30 min. Then, samples were examined with a CytoFLEX flow cytometer (Beckman Coulter, Brea, CA).

### Wound healing assay

The wound healing assay was carried out following the instructions of the manufacturer. Cells (5 × 10^5^) were seeded in a 6-well plate and a scratch wound was made with a pipette tip. After washing three times by PBS, cells were cultured in a serum-free medium and subsequently incubated in the humidified incubator at 37 °C, followed by staining using Calcein-AM (Dojindo, C326, China). Images were taken at 0, 6, 12, 24, and 48 h. The green fluorescent cells, consistent with the wound closure rate, were quantified with Image J software.

### Transcriptome analysis

Total RNA was isolated from colon tissues of each group, and coding RNA-seq libraries were prepared and sequenced by a commercial service (GENEWIZ, China). Differential expression analysis of the groups was carried out with the “EdgeR” R package with the criteria of FDR < 0.01 and |log2 fold-change (FC) | = 1. Additionally, gene set enrichment analysis (GSEA) was conducted with the application of the GSEA software (https://www.gsea-msigdb.org/, accessed on 28 January 2024).

### Detection of TGF-β1

TGF-β1 levels in colon tissue, serum, cell lysates and culture supernatants were quantified using the mouse TGF-β1 ELISA kit (Boster, EK0515, China) following the specific protocols. Optical density (OD) was determined with an EnVision 2015 Multimode Plate Reader (PerkinElmer, USA) at 37 °C.

### Statistical analysis

Experimental results were statistically explored based on GraphPad Prism 9.0.0 software. All data were shown to be mean ± SEM. Statistical differences were identified with unpaired Student’s t-test for comparisons between two groups and one-way ANOVA with Bonferroni’s post-test for comparisons between multiple groups. Differences were thought to be of significance at *p* < 0.05 (*P < 0.05; **P < 0.01; ***P < 0.001; ns, not significant).

## Supplementary information

Supplementary Information

Figure S1

Figure S2

Figure S3

The original uncropped images of western blot

WB Data and Calculation-Supplementary

## Data Availability

All relevant data supporting the main findings of this study are available in the article and its *Supplementary Information files*. Any additional information required to reanalyze the data reported in this paper is available from the lead contact upon request.
