# Supplementary material for: SAA3 deficiency exacerbates intestinal fibrosis in DSS-induced IBD mouse model
Source: Cell Death Discov. 2025 Jan 26;11:25. doi: 10.1038/s41420-025-02299-x (PMC11763003; doi:10.1038/s41420-025-02299-x)
Supplement: Supplementary file 1 — Supplementary Information [file 41420_2025_2299_MOESM1_ESM.docx]

**Supplementary Information**

**Table 1**: The primary and secondary antibodies are listed in table.

| **Antibodies** | **Cat No.** | **Host** | **Supplier** |
| --- | --- | --- | --- |
| TGF Beta 1 Polyclonal antibody | 21898-1-AP | Rabbit | Proteintech |
| smooth muscle actin Polyclonal antibody | 14395-1-AP | Rabbit | Proteintech |
| COL1A1(C-terminal propeptide) Antibody | P28372-B1 | Rabbit | Abmart |
| NF-κB p65 (D14E12) XP® Rabbit mAb | 8242 | Rabbit | Cell Signaling Technology |
| Phospho-NF-κB p65 (Ser536) (93H1) Rabbit mAb | 3033 | Rabbit | Cell Signaling Technology |
| Recombinant Anti-SAA3 antibody | ab233547 | Rabbit | Abcam |
| HSP27 Polyclonal antibody | 18284-1-AP | Rabbit | Proteintech |
| Phospho-HSP27 (Ser78) Polyclonal antibody | 28900-1-AP | Rabbit | Proteintech |
| IκBα Antibody | 9242 | Rabbit | Cell Signaling Technology |
| Phospho-IκBα (Ser32) (14D4) Rabbit mAb | 2859 | Rabbit | Cell Signaling Technology |
| SMAD2 Polyclonal antibody | 12570-1-AP | Rabbit | Proteintech |
| Phospho-SMAD2 (Ser465/467) (138D4) Rabbit mAb | 3108 | Rabbit | Cell Signaling Technology |
| SMAD3 Monoclonal antibody | 66516-1-Ig | Rabbit | Proteintech |
| Phospho-SMAD3 (Ser423/425) (C25A9) Rabbit mAb | 9520 | Rabbit | Cell Signaling Technology |
| Beta Actin Monoclonal antibody | 66009-1-Ig | Mouse | Proteintech |
| HRP-conjugated Affinipure Goat Anti-Mouse IgG(H+L) | SA00001-1 | Goat | Proteintech |
| HRP-conjugated Affinipure Goat Anti-Rabbit IgG(H+L) | SA00001-2 | Goat | Proteintech |

**Table 2**: List of primers used for qRT-PCR.

| **Primers** | **Sequences (5' to 3')** | **Amplicon (bp)** |
| --- | --- | --- |
| Mouse-TGF-β1 | CCACCTGCAAGACCATCGAC | 91 |
|  | CTGGCGAGCCTTAGTTTGGAC |  |
| Mouse-Col1a1 | CGACCTCAAGATGTGCCACT | 219 |
|  | CCATCGGTCATGCTCTCTCC |  |
| Mouse-α-SMA | CCTTCGTGACTACTGCCGAG | 243 |
|  | TATAGGTGGTTTCGTGGATGCC |  |
| Mouse-Slc1a4 | TTGCTTACTTCGGCCTCACC | 220 |
|  | GACGTAGTGAATGCGGCAAC |  |
| Mouse-Gm45629 | TTTGGTCACTGACTCGAGGC | 174 |
|  | CAGGGACTTGAAGCTCAGCA |  |
| Mouse-Hspb1 | ATGAGTGGTCGCAGTGGTTC | 266 |
|  | TTCGTGCTTGCCAGTGATCT |  |
| Mouse-Cphx1 | CATACGCTCCTTACCCGGAC | 154 |
|  | CTGCGCATTCTTCTCATGGC |  |
| Mouse-Saa3 | TGAAGCCTTCCATTGCCATC | 115 |
|  | TAGGCTCGCCACATGTCTCT |  |
| Mouse-Tmem254 | CCCTAGGGCTGGGCTACTTC | 268 |
|  | ATGGAGAGAGAGGCTACACC |  |
| Mouse-Msmo1 | TCCTCTCAACCCGCTGAACT | 135 |
|  | TACTGGGCATCTGTCCCAAAG |  |
| Mouse-β-actin | AGCCATGTACGTAGCCATCC | 228 |
|  | CTCTCAGCTGTGGTGGTGAA |  |


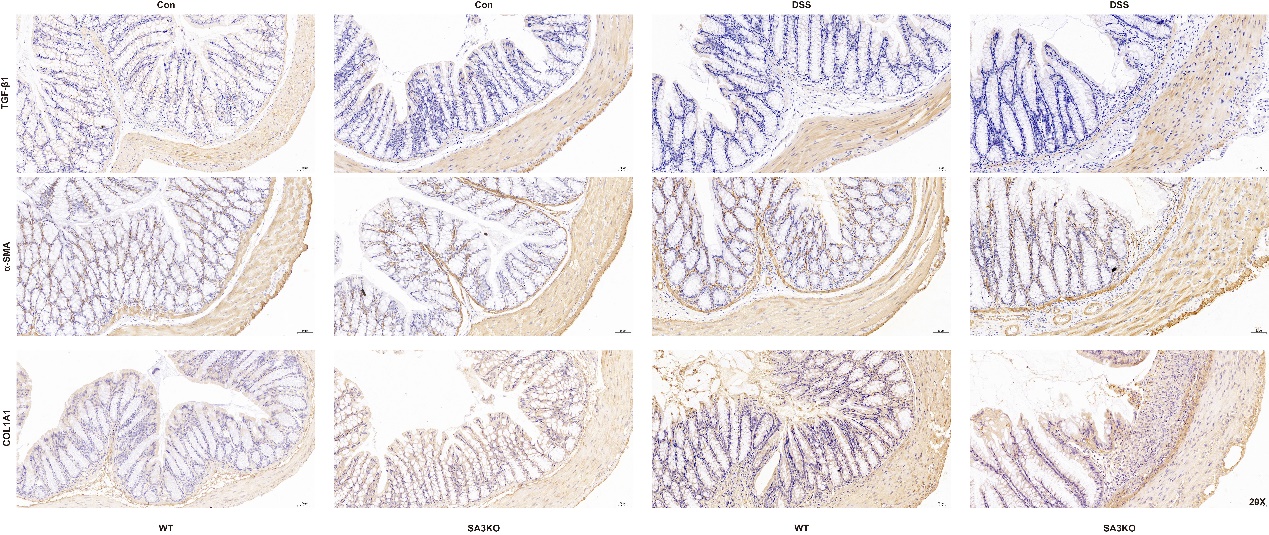


**Figure S1.** Representative images of IHC-stained colon sections for TGF-β1, α-SMA, and COL1A1 from the control and DSS-treated mice after 10 weeks.


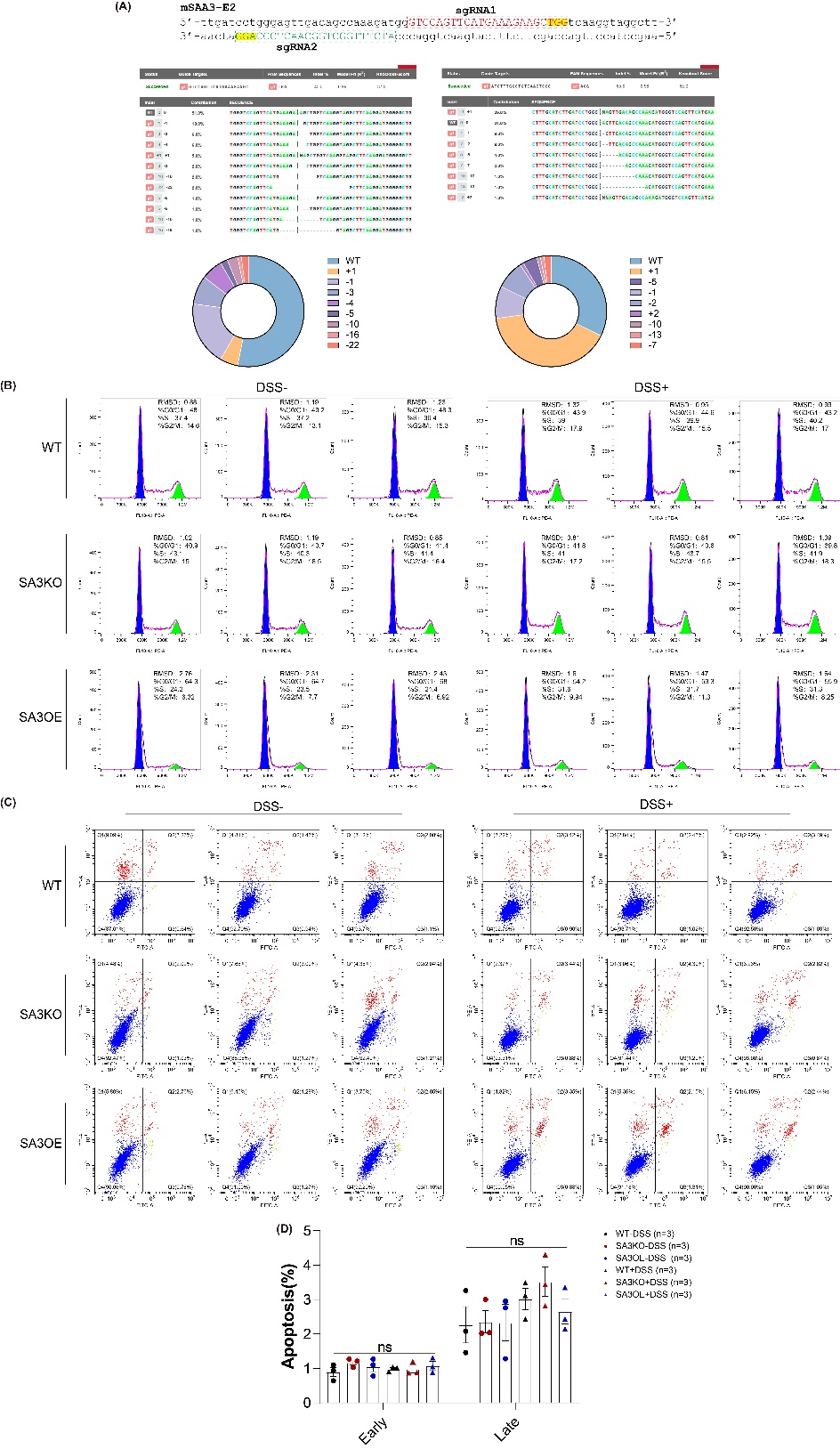


**Figure S2.** (A) Construction and efficiency evaluation of the SAA3 gene editing system. (B) Detection of cell cycle. N=3, biological replicates. (C) Detection of cell apoptosis. N=3, biological replicates; ns, not significant.


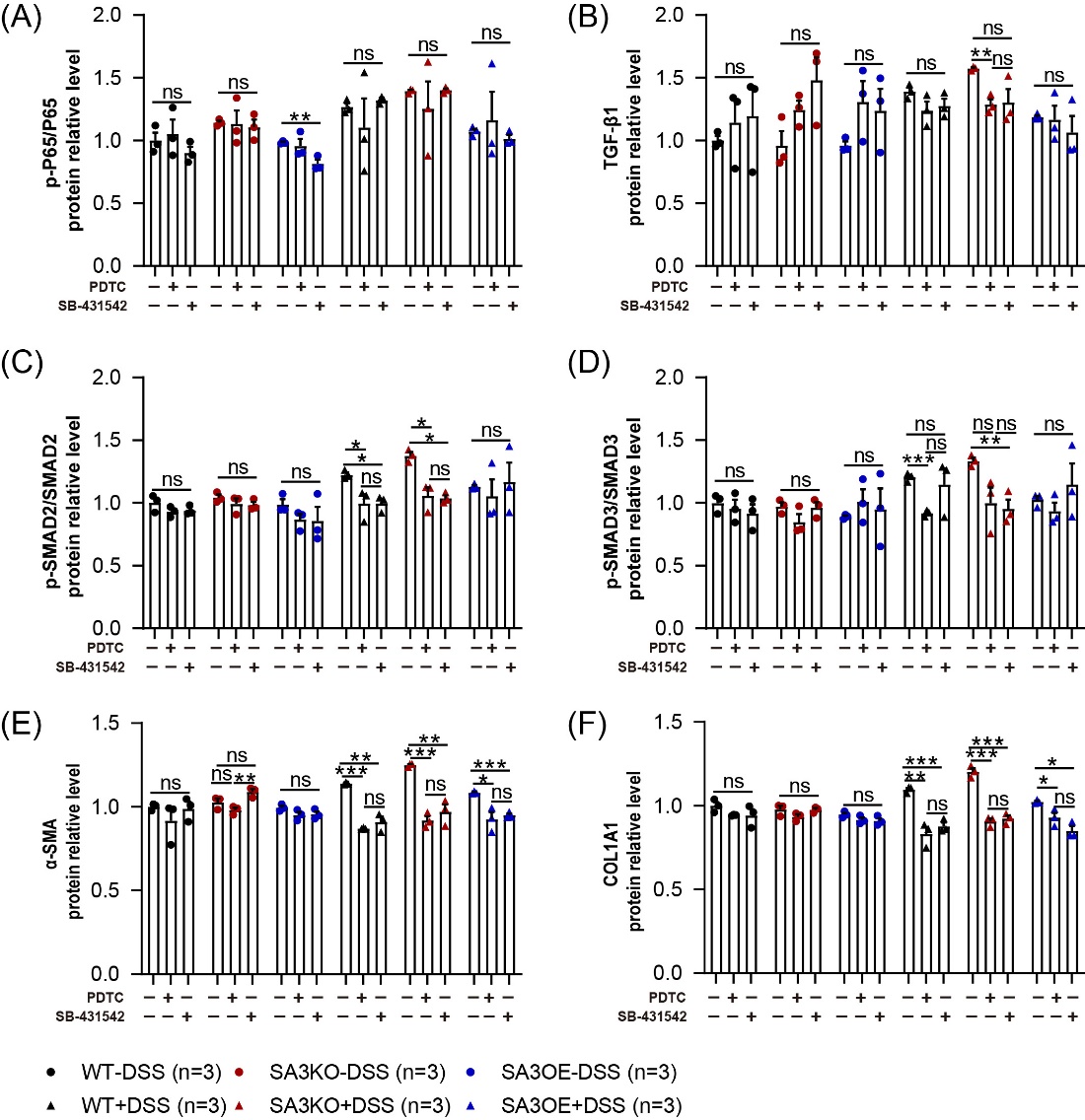


**Figure S3.** Changes in NF-κB, TGF-β1, and Smad signaling molecules were detected by WB. N=3, biological replicates; ns, not significant; *P<0.05; **P<0.01; ***P<0.001.
