# Supplementary figures and images for: SAA3 deficiency exacerbates intestinal fibrosis in DSS-induced IBD mouse model
Source: Cell Death Discov. 2025 Jan 26;11:25. doi: 10.1038/s41420-025-02299-x (PMC11763003; doi:10.1038/s41420-025-02299-x)

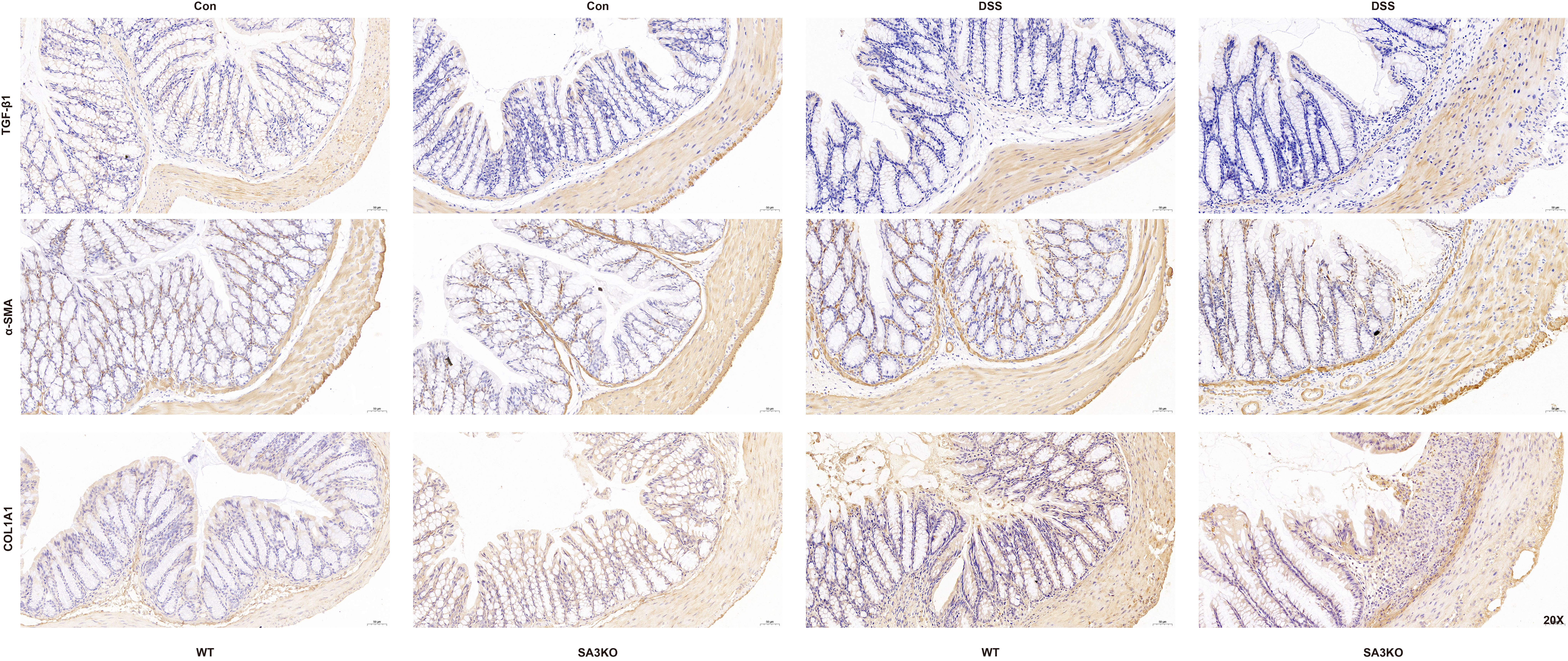

Supplement: Supplementary file 2 — Figure S1 [file 41420_2025_2299_MOESM2_ESM.jpg]

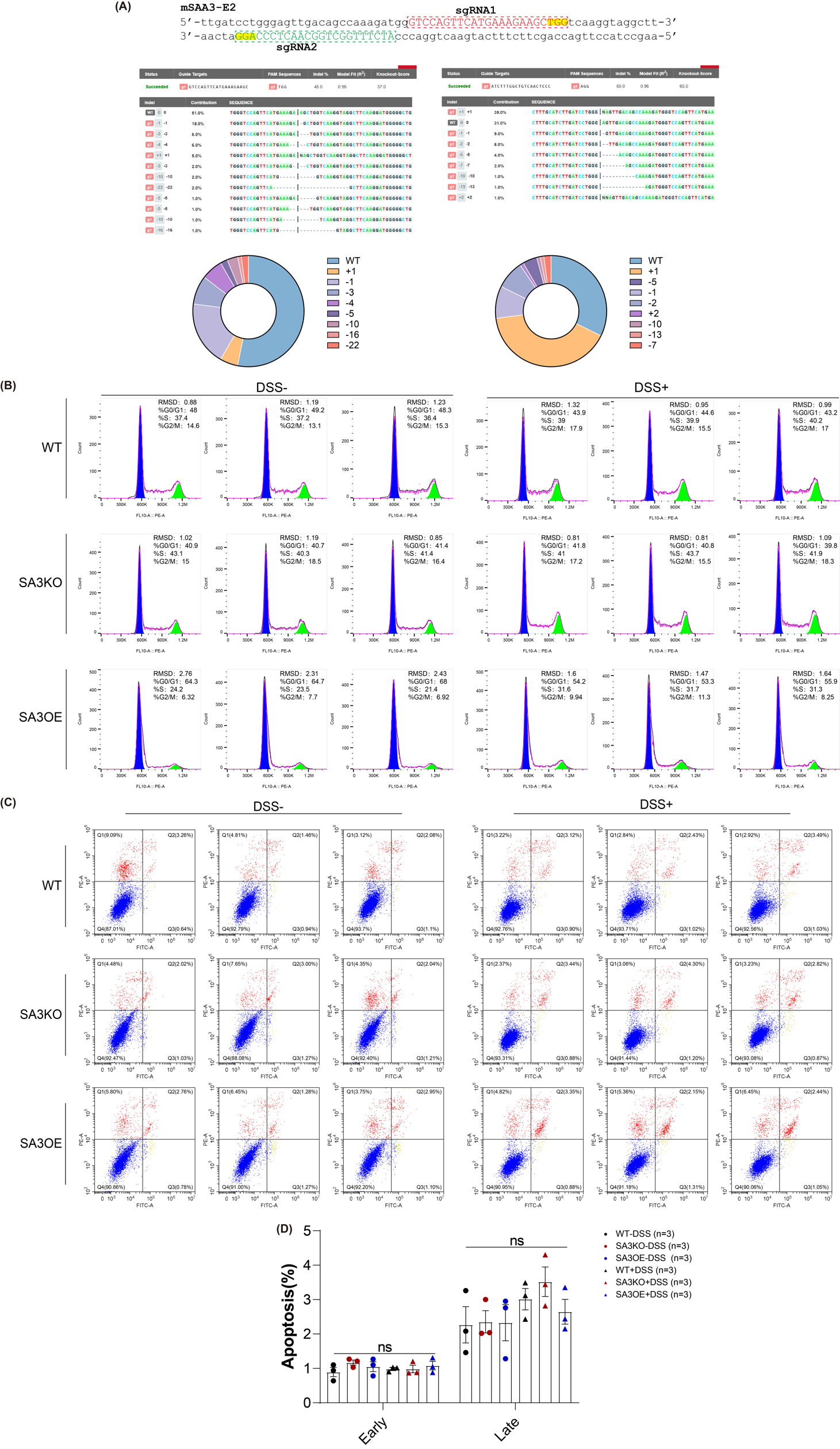

Supplement: Supplementary file 3 — Figure S2 [file 41420_2025_2299_MOESM3_ESM.jpg]

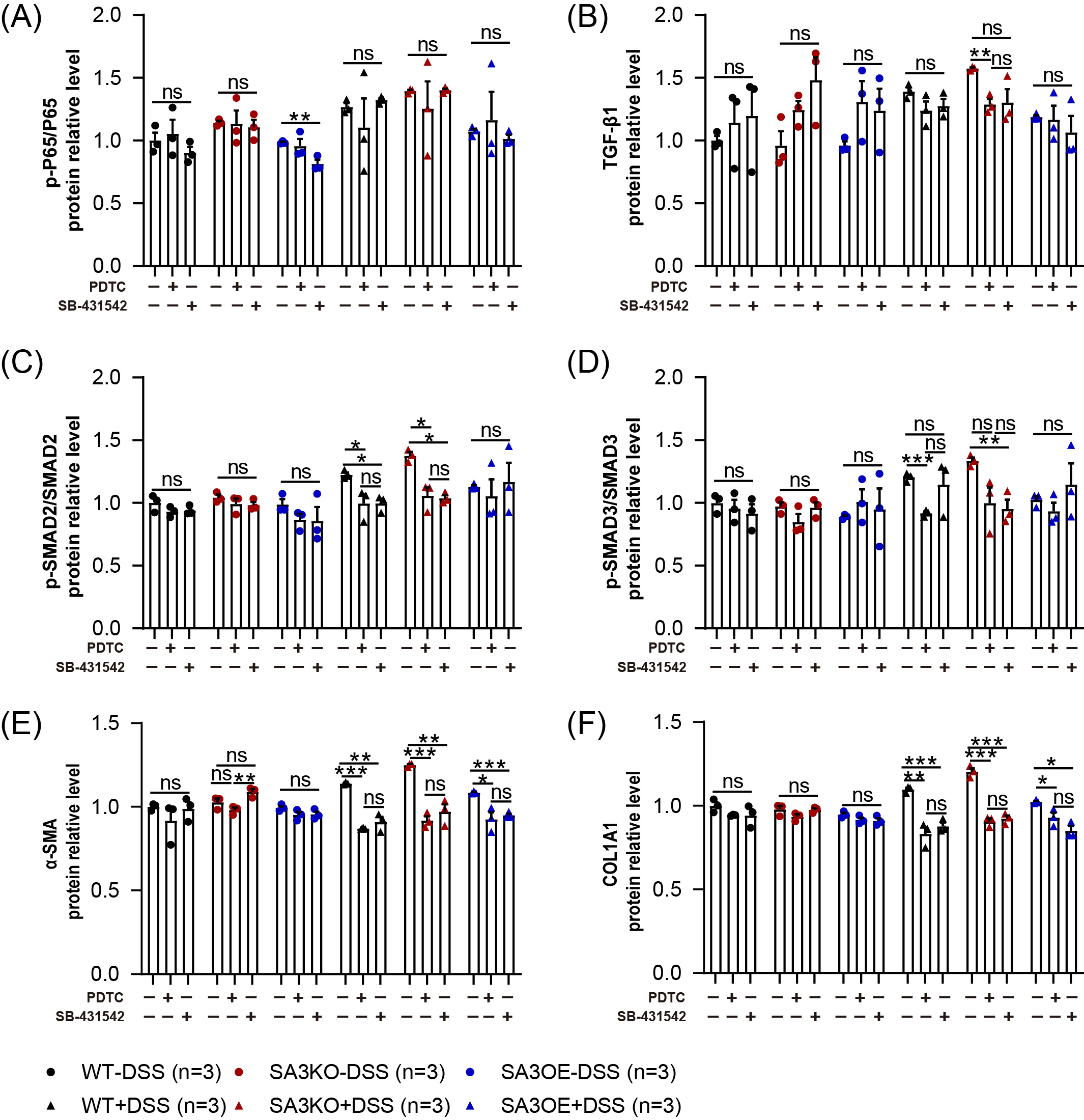

Supplement: Supplementary file 4 — Figure S3 [file 41420_2025_2299_MOESM4_ESM.jpg]
