# Supplementary material for: SAA3 deficiency exacerbates intestinal fibrosis in DSS-induced IBD mouse model
Source: Cell Death Discov. 2025 Jan 26;11:25. doi: 10.1038/s41420-025-02299-x (PMC11763003; doi:10.1038/s41420-025-02299-x)

F2F

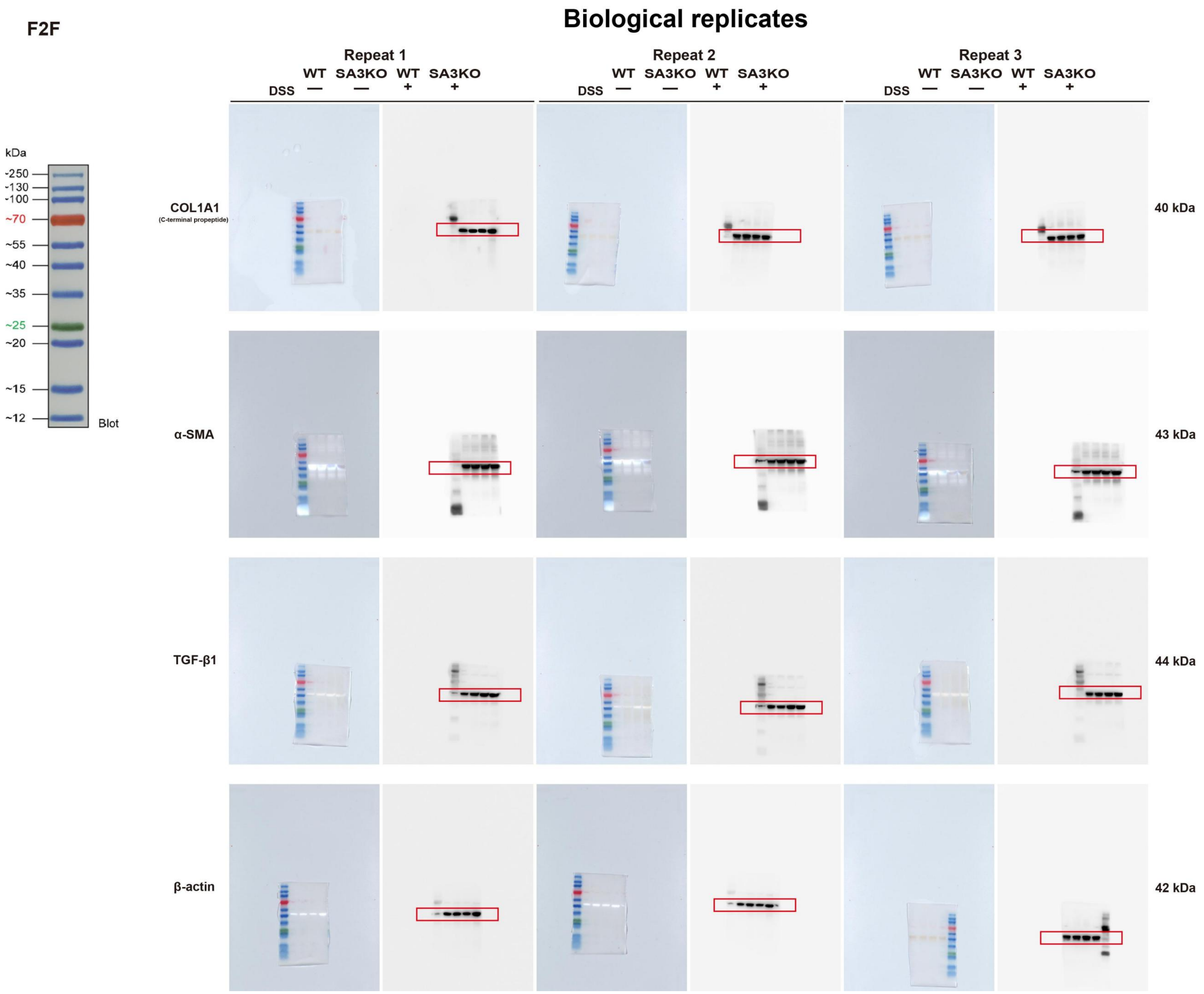
Biological replicates
Repeat 1
Repeat 2
Repeat 3
DSS
WT
SA3KO
—
+
kDa
~250
~130
~100
~70
~55
~40
~35
~25
~20
~15
~12
Blot
COL1A1
(C-terminal propeptide)
α-SMA
TGF-β1
β-actin
40 kDa
43 kDa
44 kDa
42 kDa

F3F

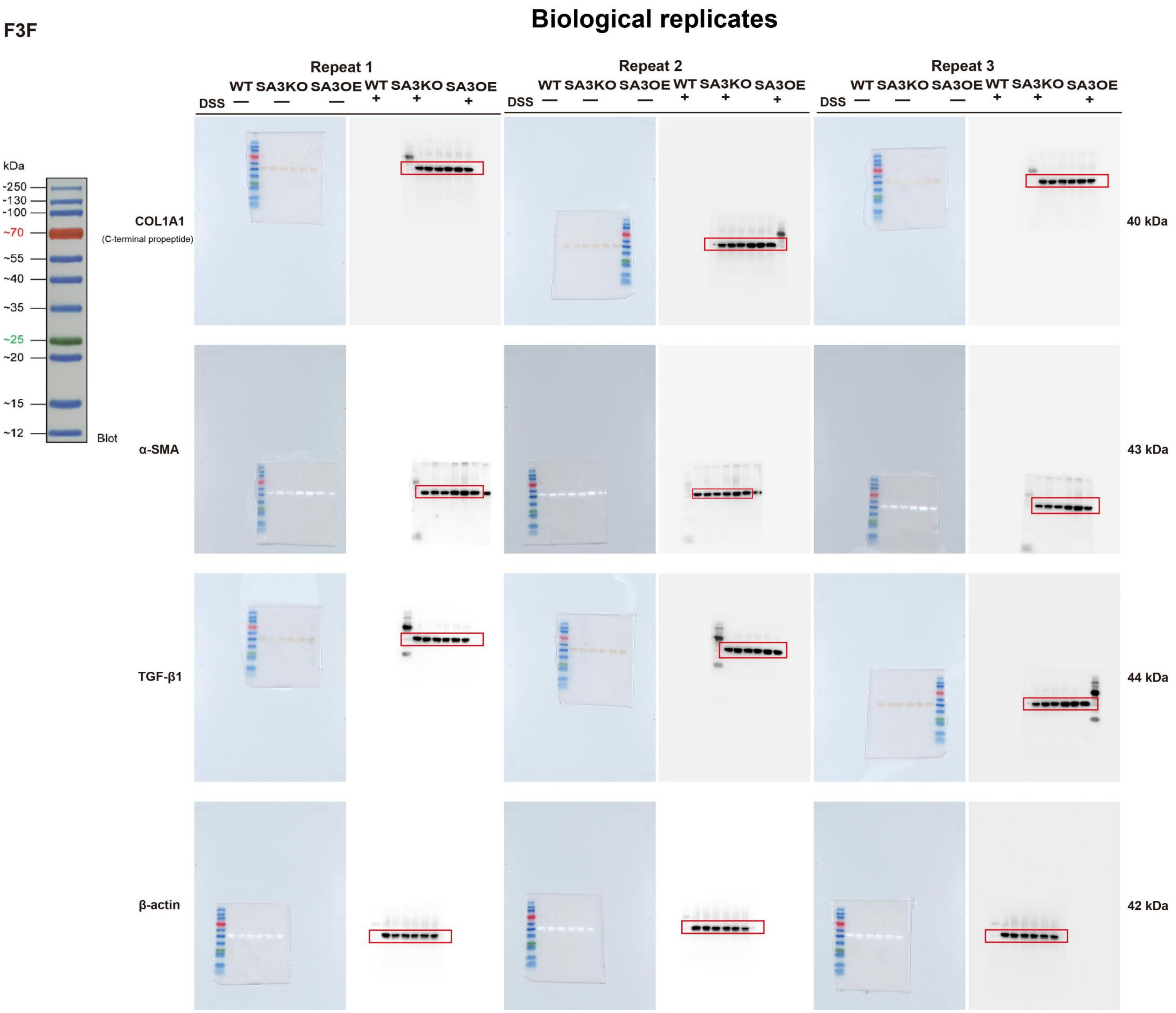

Biological replicates
Repeat 1
Repeat 2
Repeat 3
WT SA3KO SA3OE WT SA3KO SA3OE
DSS — — — + + +
kDa
~250
~130
~100
~70
~55
~40
~35
~25
~20
~15
~12
Blot
COL1A1
(C-terminal propeptide)
α-SMA
TGF-β1
β-actin
40 kDa
43 kDa
44 kDa
42 kDa

F4C

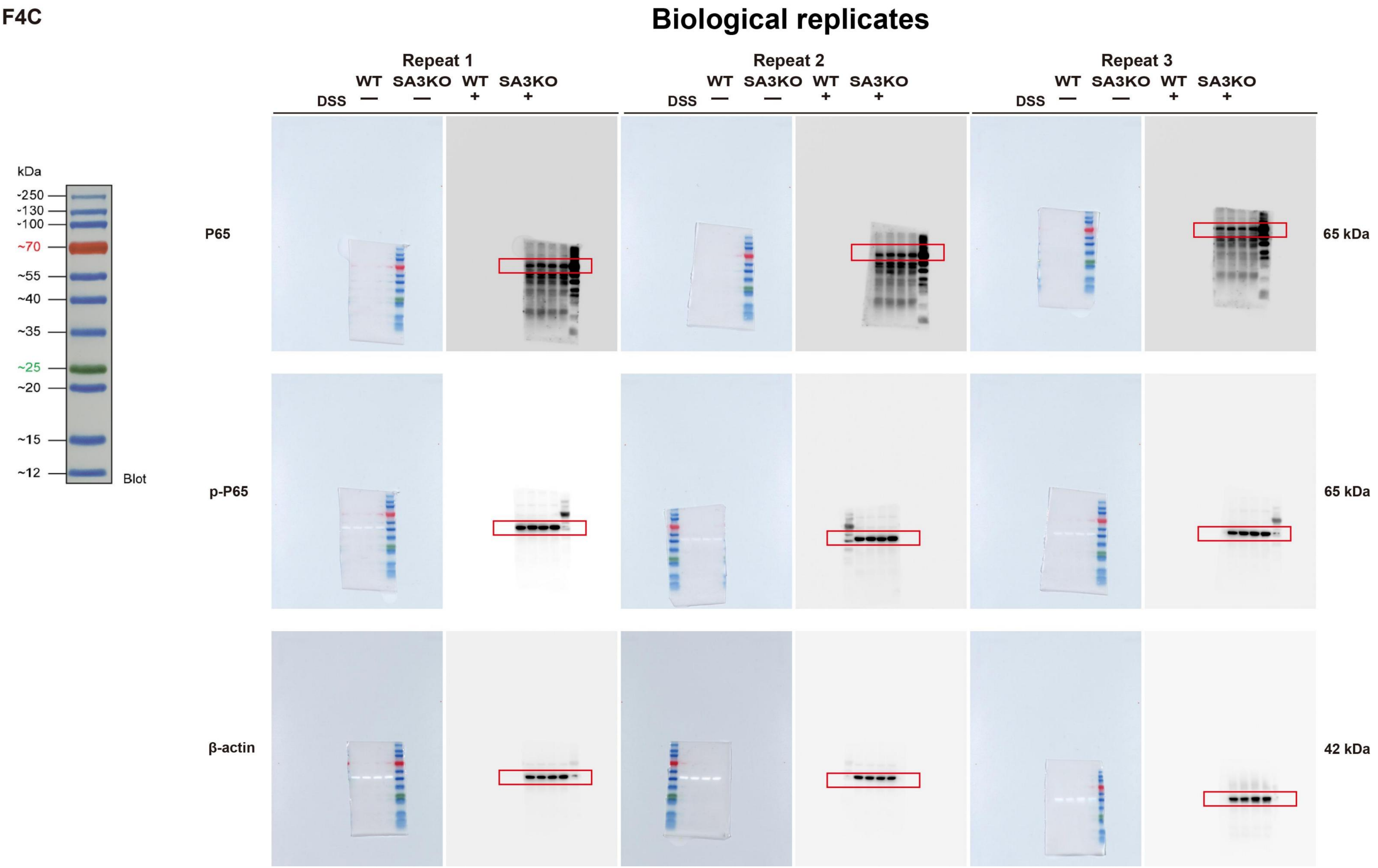

Biological replicates
Repeat 1
Repeat 2
Repeat 3
DSS
WT
SA3KO
WT
SA3KO
—
—
+
+
P65
p-P65
β-actin
65 kDa
65 kDa
42 kDa
kDa
~250
~130
~100
~70
~55
~40
~35
~25
~20
~15
~12
Blot

F5C

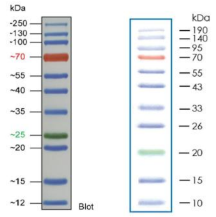
kDa
~250
~130
~100
~70
~55
~40
~35
~25
~20
~15
~12
Blot
kDa
190
140
95
70
55
43
33
26
20
15
10

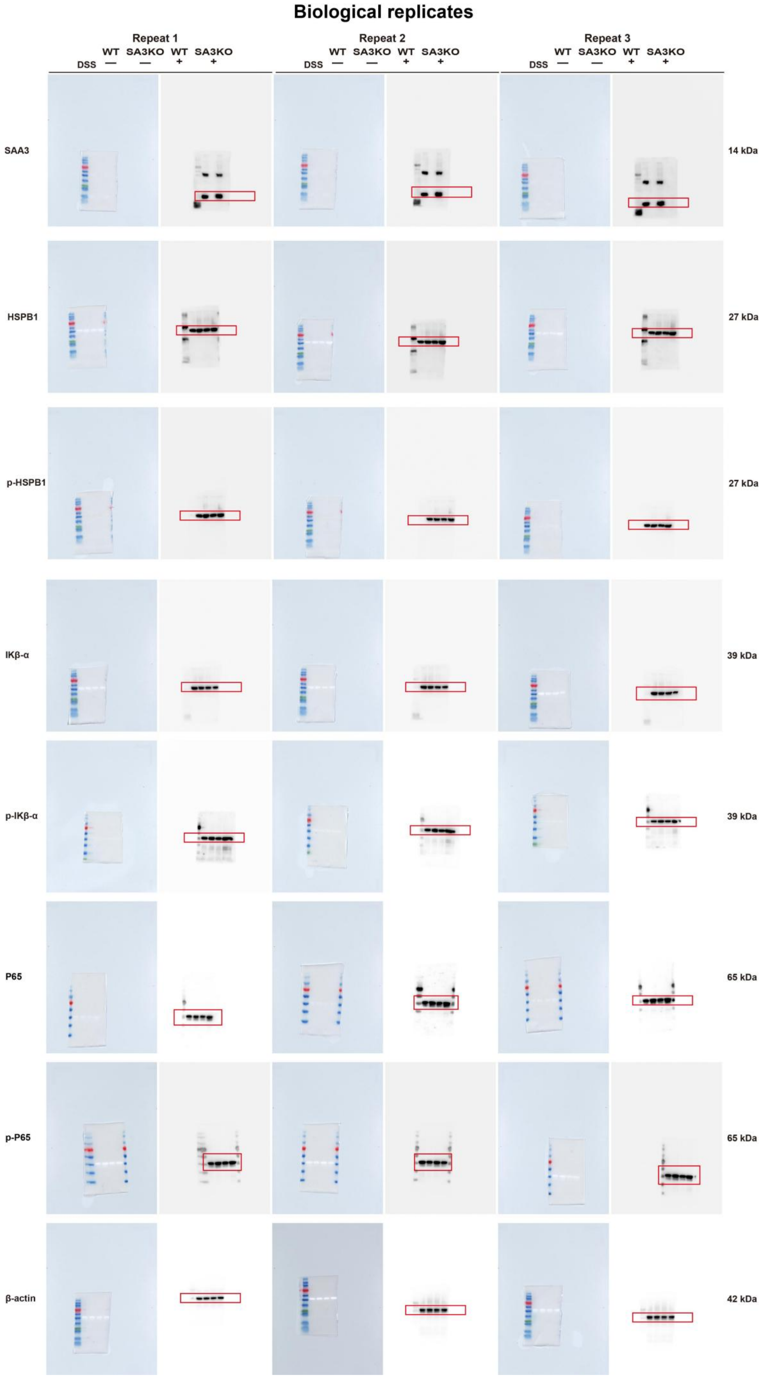
Biological replicates
Repeat 1
Repeat 2
Repeat 3
DSS
WT
SA3KO
WT
SA3KO
—
—
+
+
SAA3
14 kDa
HSPB1
27 kDa
p-HSPB1
27 kDa
IKβ-α
39 kDa
p-IKβ-α
39 kDa
P65
65 kDa
p-P65
65 kDa
β-actin
42 kDa

F5D

Biological replicates

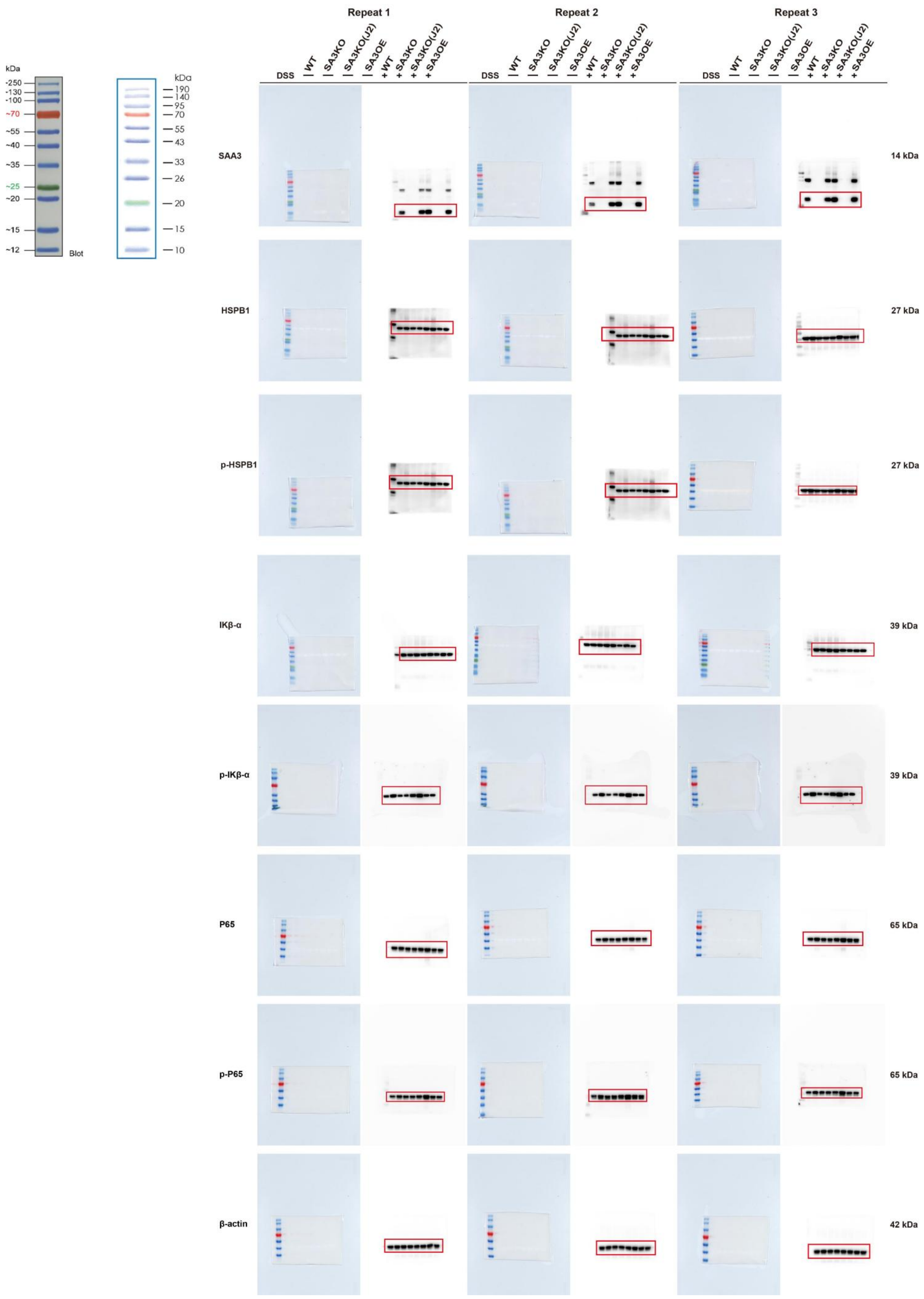

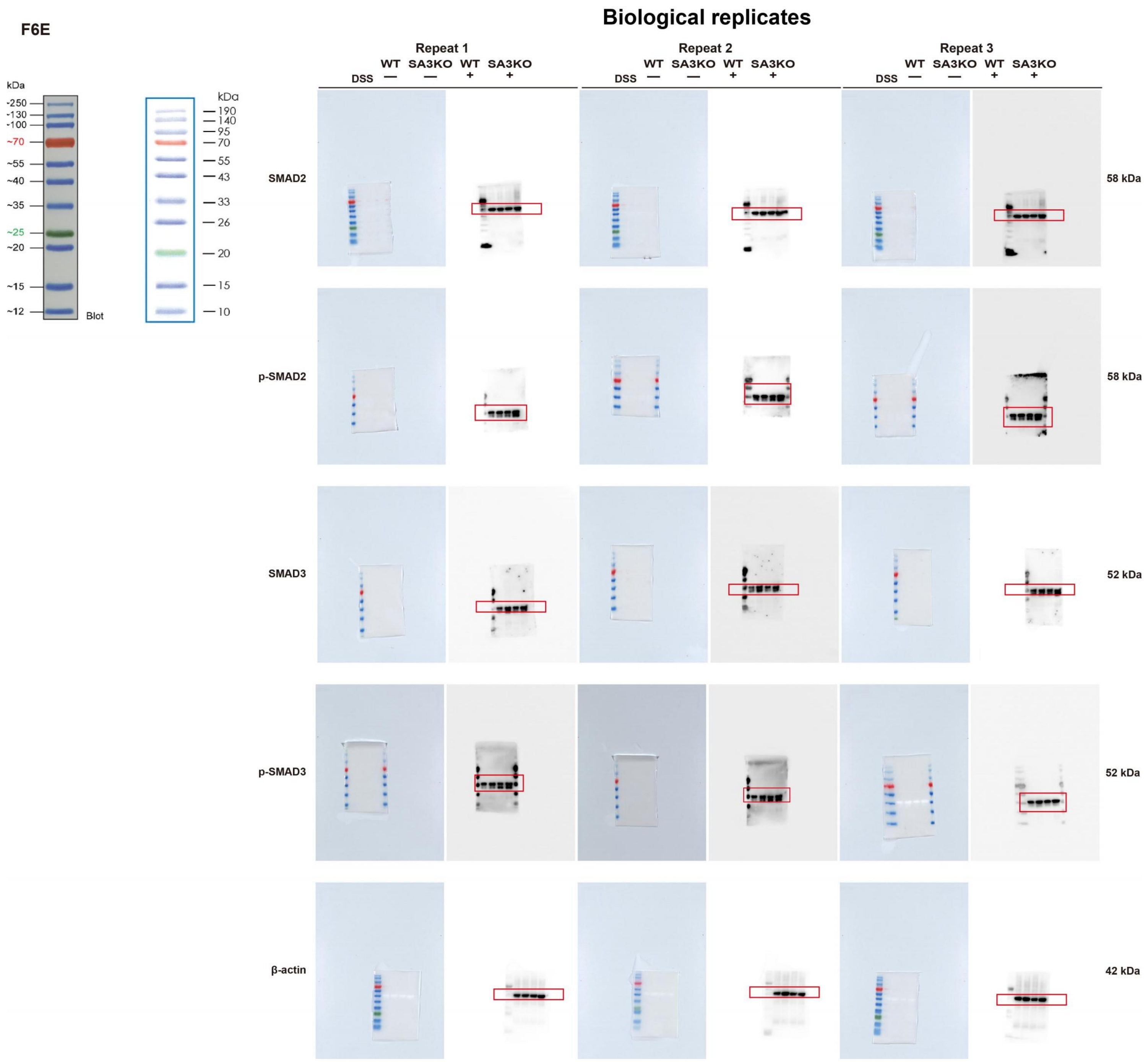
F6E
kDa
~250
~130
~100
~70
~55
~40
~35
~25
~20
~15
~12
Blot
kDa
190
140
95
70
55
43
33
26
20
15
10
Biological replicates
Repeat 1
Repeat 2
Repeat 3
DSS
WT
SA3KO
WT
SA3KO
—
—
+
+
SMAD2
p-SMAD2
SMAD3
p-SMAD3
β-actin
58 kDa
58 kDa
52 kDa
52 kDa
42 kDa

F6F-1

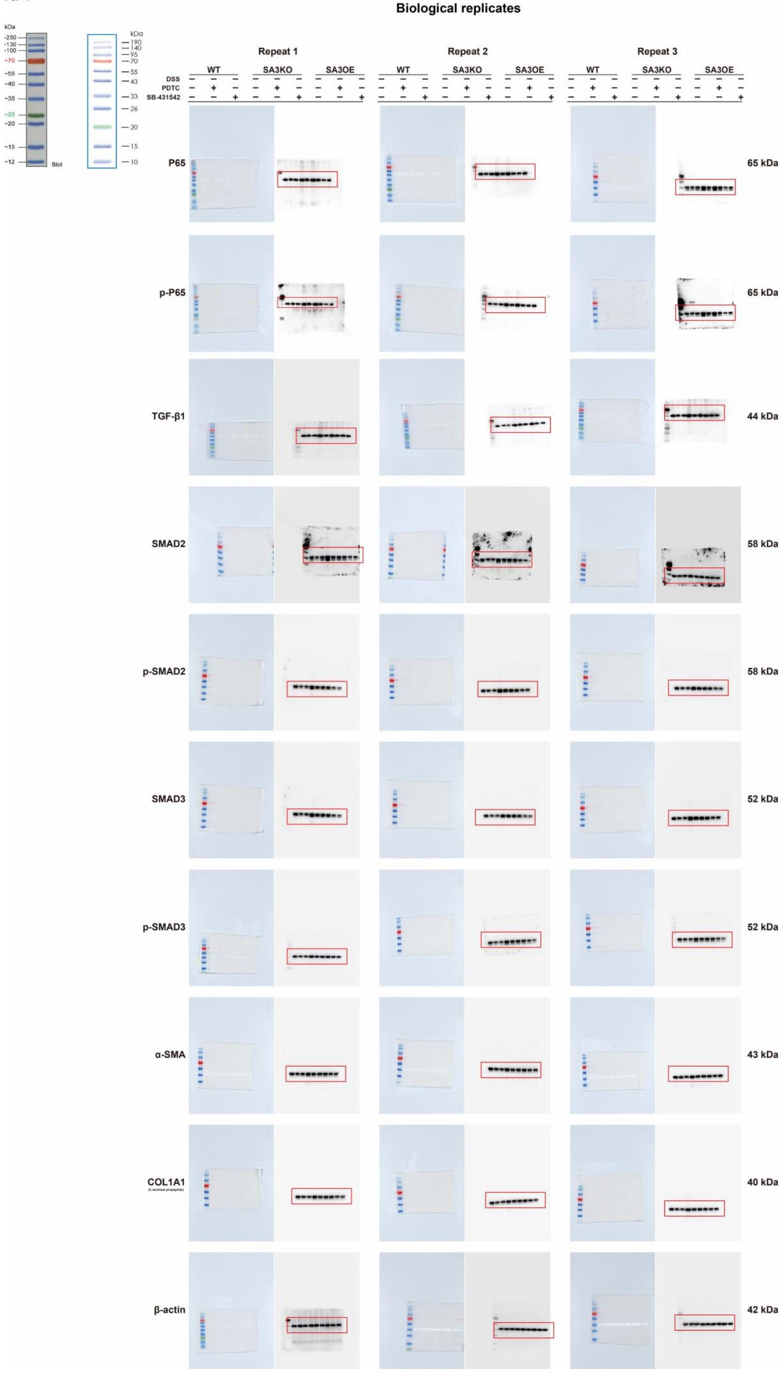

Biological replicates
Repeat 1
Repeat 2
Repeat 3
WT
SA3KO
SA3OE
DSS
PDTC
SB-431542
P65
65 kDa
p-P65
65 kDa
TGF-β1
44 kDa
SMAD2
58 kDa
p-SMAD2
58 kDa
SMAD3
52 kDa
p-SMAD3
52 kDa
α-SMA
43 kDa
COL1A1
40 kDa
β-actin
42 kDa

F6F-2

Biological replicates

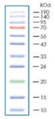

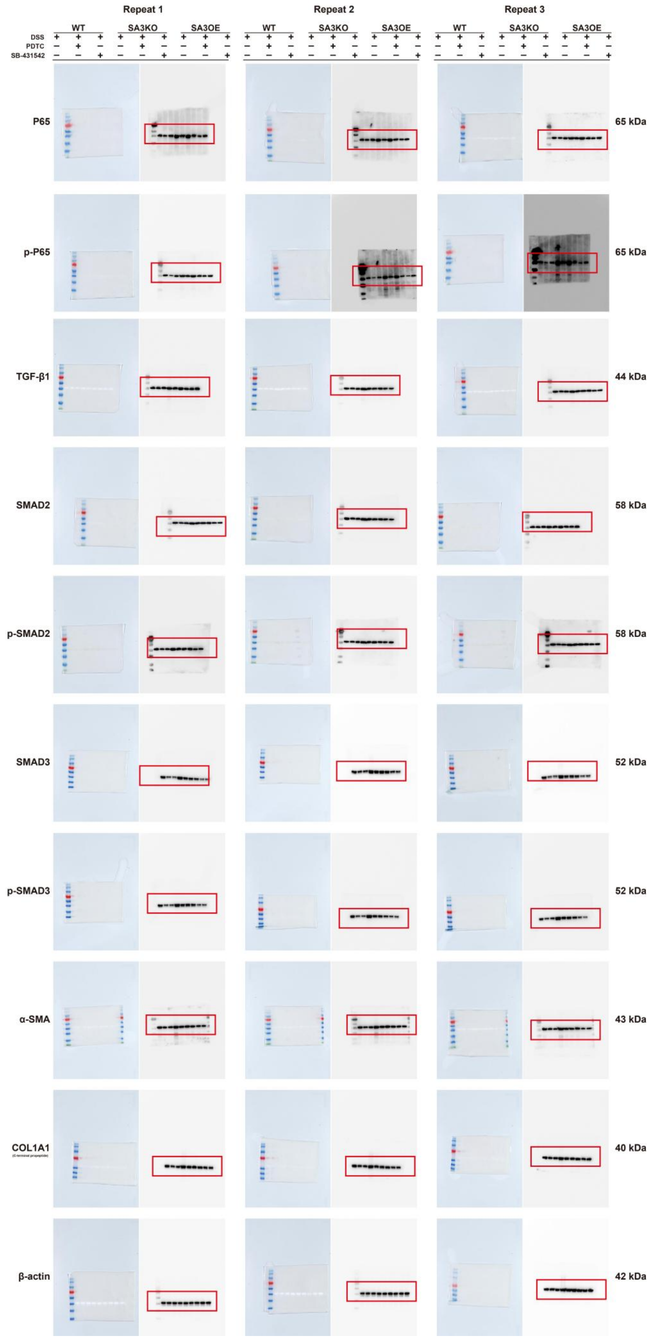

Supplement: Supplementary file 5 — The original uncropped images of western blot [file 41420_2025_2299_MOESM5_ESM.pdf]
